# Sacrificial Methionine Residues Protect Active-Site Histidines From Oxidation

**DOI:** 10.1002/anov.70034

**Published:** 2026-09-17

**Authors:** Evans C. Wralstad, Jessica Sayers, Giulio Fittolani, David Sarabia-Castillo, Alex J. Callahan, Bradley L. Pentelute, Ronald T. Raines

**Affiliations:** Department of Chemistry, Massachusetts Institute of Technology, Cambridge, Massachusetts, USA

**Keywords:** histidine, methionine, oxidative damage, protein synthesis, ribonuclease

## Abstract

Histidine is the most common residue in enzymic active sites because of the chemical versatility of its imidazole side chain, but is also highly vulnerable to oxidative damage. Here, we demonstrate that methionine residues in secretory ribonucleases such as bovine RNase A serve as sacrificial antioxidants that protect active-site histidines. In the human homolog RNase 1, five methionine residues cluster near the catalytic histidines His12 and His119, which serve as a base and an acid, respectively, during RNA cleavage. Bioinformatic analyses reveal that these methionines are conserved across the RNase A superfamily and adopt *S*–aryl motifs with proximal aromatic residues that modulate the electron density at sulfur and thereby tune the susceptibility of methionine to oxidation. Chemoselective labeling of methionine with an oxaziridine probe increased the vulnerability of RNase 1 to oxidation-induced inactivation, implicating Met29 and Met35 as protective residues. Total chemical synthesis of an RNase 1 variant in which these residues were replaced by norleucine, a sulfur-free isostere of methionine, corroborated their protective role. A similar arrangement of methionines is present in unrelated secretory proteins, including serine proteases and calprotectin, suggesting a broadly recurring protective strategy.

## EDITORIAL SUMMARY

Enzymes carry out the chemistry of life, but the key amino acids that drive reactions in their active sites are vulnerable to damage by reactive oxygen species. Histidine, the most common residue in enzyme active sites, is a prime example: its oxidation is irreversible and switches the enzyme off, raising the long-standing question of how enzymes function in harsh, oxygen-rich environments. Combining bioinformatic analysis with chemoselective labeling and total chemical synthesis, Raines and co-workers show that a pair of neighboring methionine residues in human ribonuclease 1 act as sacrificial shields, absorbing oxidative damage to spare the vital histidines, and that the same protective arrangement recurs across unrelated proteins. The Scientific Advisory Board selected this study for Angewandte Chemie Novit because it reveals an elegant, broadly conserved strategy for surviving oxidative stress that had remained hidden in plain sight within one of the most-studied proteins in chemistry.

## Graphical Abstract

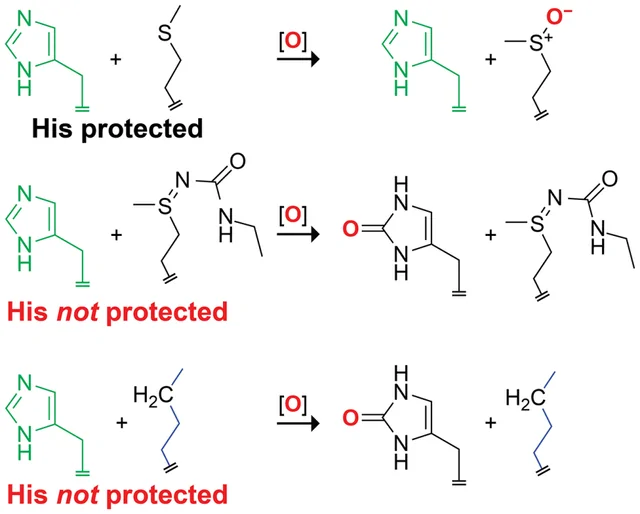

## 1 | Introduction

Among the twenty canonical amino acids, cysteine and methionine are relatively rare residues that are well known for their susceptibility to oxidation by reactive oxygen species (ROS). Histidine is the most common residue in enzymic active sites [1, 2] and is often critical for other biochemical functions [3, 4]. Notably, after cysteine and methionine, His is the most reactive residue with ROS [5]. This vulnerability arises because the electron-rich imidazole ring is susceptible to electrophilic attack at C2, producing 2-oxohistidine. The product, 2-oxohistidine, is structurally analogous to 2-oxoguanine, the most prevalent oxidatively damaged nucleobase (Figure 1A) [6].

Secretory ribonucleases (RNases) constitute a diverse class of enzymes that catalyze the cleavage of RNA. The predominant secretory ribonucleases in vertebrates are members of the RNase A superfamily. These enzymes are among the most thoroughly characterized proteins [7–10] and are involved in digestion, angiogenesis, host defense, extracellular RNA metabolism, and other physiological processes [10–16].

Catalysis of RNA cleavage by RNase A relies upon two renowned histidine residues [17, 18]. These residues are conserved throughout the superfamily, including in RNase 1, which is the prevalent human homolog and circulates at 0.5 μg mL^−1^ (0.04 μM) in serum [19]. In its catalytic mechanism (Figure 1B), His12 acts as a base, deprotonating the 2′-hydroxy group of a ribonucleotide to generate a nucleophilic alkoxide that attacks the adjacent 3′-phosphodiester. His119 acts as an acid, protonating the 5′-oxygen leaving group to facilitate P─O bond scission (Figure 1B). The requisite protonation states of these histidine residues give rise to the archetypal bell-shaped pH–rate profile for catalysis [20], and replacing either histidine with alanine decreases catalytic efficiency (*k*_cat_/*K*_M_) by 10^4^-fold [21, 22]. Meanwhile, Lys41 and Phe120 orient and polarize the anionic phosphoryl group [7].

Unlike intracellular proteins, secretory proteins must maintain their function in the relatively oxidizing environment of the extracellular space, including serum [23]. This requirement is especially challenging for proteins with oxidation-prone residues, such as RNase A and RNase 1. Indeed, histidine oxidation has long been known to result in the loss of their ribonucleolytic activity [24], in line with aforementioned alanine mutagenesis experiments.

We observed that RNase 1 contains five methionine residues, nearly twice the expected number for a human protein of its size. These residues cluster near the active site and appear poised to act as sacrificial antioxidants that protect the critical histidine residues (Figure 1C). The thioether sulfur of methionine is a soft nucleophile that reacts readily with ROS such as the hydroxyl radical to form the corresponding sulfoxide, a thermodynamically favorable and kinetically competitive transformation relative to the oxidation of histidine [24]. A protective methionine “phalanx” has been proposed to exist in other proteins [25–28] but without experimental verification that goes beyond demonstrating that methionine residues undergo oxidation.

Here, we report experimental evidence that methionine residues confer oxidative resistance to the active site of RNase 1. We use bioinformatic analyses to interrogate the evolutionary patterns of methionine usage in the RNase A superfamily and identify structural motifs that suggest a residue-specific tuning of methionine reactivity. We then use two orthogonal chemical strategies to perturb the antioxidative capacity of specific methionine residues: chemoselective labeling with an oxaziridine and total chemical synthesis incorporating a sulfur-free isostere. Both approaches diminish the ability of RNase 1 to withstand oxidation. Finally, we present evidence that our results with RNase 1 may be relevant to the function of other secretory proteins that rely on histidine residues. Broadly, these findings support an underappreciated role for the thioether sulfur of methionine in maintaining biological function under oxidative stress—one that was hiding in plain sight in one of the most-studied proteins in chemistry.

## 2 | Results and Discussion

### 2.1 | Methionine Conservation in Homologs

To assess whether the methionine residues near the RNase 1 active site are evolutionarily conserved, and therefore likely functional, we used the Dali protein structure comparison server [29] to identify 19 non-redundant homologs across vertebrate species with RNase 1 sequence identity ranging from 24%–72% (Figure S1A). Despite this broad range of sequence identity, all 19 proteins are structurally similar (Figure S1B). We surveyed our homolog set for patterns of methionine conservation relative to RNase 1 and determined the frequency of conserved residue solvent accessibility and the average distance of each conserved methionine to the strictly conserved active-site residues homologous to His12 and His119 (Table S1). The data reveal several trends: (1) methionines aligned to RNase 1 Met29 and Met30 are frequently present, (2) methionines aligned to Met30 and Met35 are consistently proximal to active-site residues, and (3) methionines aligned to Met29 and Met35 are frequently exposed to solvent. Structural alignment of the homolog set revealed that, despite the conformational flexibility of the methionine side chain, conserved methionines adopt consistent conformations with retention of the orientation of the methionyl sulfur atom (Figure S2).

Phylogenetic analysis revealed that methionine content increases during the evolution of the RNase A superfamily, from the more ancestral RNase 5 to the more descendant enzymes (Figure S3) [30], which are believed to have arisen through gene duplication [13, 31, 32]. Greater methionine utilization correlates with higher ribonucleolytic activity [14, 32], suggesting that methionine incorporation has been selected for in parallel with catalytic efficiency.

### 2.2 | *S*–Aryl Motifs in Homologs

We next asked whether the 3D structures of RNase 1 homologs contain features that could modulate the oxidative reactivity of the conserved methionine residues. A logo plot of the aligned homolog set sequences reveals that several aromatic residues are highly conserved, some nearly as strictly as the active-site and half-cystine residues (Figure S4) [33, 34]. Sulfur–arene interactions, referred to as the “*S*–aryl motif” in the context of proteins, are a significant secondary force in proteins with interaction energies of 1–3 kcal mol^−1^, on par with salt bridges [35–39]. In these motifs, the polarizable sulfur atom of methionine engages in a noncovalent interaction with the *π*-electron system of an aromatic ring. The geometry and directionality of this interaction determine whether electron density is donated to or withdrawn from sulfur, which in turn should modulate the ease with which the thioether is oxidized. We hypothesize that the conservation of aromatic residues in our homolog set is related to patterns of methionine conservation.

To screen for *S*–aryl motifs in the structures of our homolog set, we applied a 7-Å cut-off on sulfur–arene distance (Figure S5) [37]. All of these *S*–aryl motifs are vicinal to the active site and highly conserved among homologs. We characterized these *S*–aryl motifs based on (1) the distance (*d*) between the methionyl sulfur atom and the nearest aromatic ring atom, (2) the angle (*θ*) of approach between the methionyl sulfur and the plane of the aromatic ring, and (3) whether the methionyl thioether group was oriented such that the sulfur atom or the thioether carbon atoms were pointed toward the aromatic ring (“*S*-toward” and “*S*-away”, respectively). The *S*-toward interaction directs sulfur lone pair electrons toward the aromatic ring and is best complemented by the partially electron-deficient (*δ*^+^) ring edge, whereas the *S*-away interaction directs methionyl C─S σ* orbitals toward the aromatic ring and is best complemented by the electron-rich (*δ*^−^) ring faces [36]. As shown in Figure 2, the sulfur orientation and angle of approach largely complement one another for the *S*–aryl motifs present in our homolog set: approach angles <30° (i.e., approach along the ring edge) tend to correlate with an *S*-toward orientation, and approach angles >70° (i.e., approach along the ring face) tend to correlate with an *S*-away orientation. Of the four methionines involved in *S*–aryl motifs, Met29 is engaged in two interactions (with Tyr25 and Phe46) that direct electron density into the thioether; Met35 is engaged in one donating interaction (with Phe46) and in one abstracting interaction (albeit with the active-site residue His12); Met30 is engaged in one donating interaction (with Tyr97) that is offset by an even closer abstracting interaction (with Phe46); and Met13 is engaged in one abstracting interaction (with Phe8). We hypothesize that *S*–aryl motifs that donate electron density to the methionyl sulfur atom should increase its nucleophilicity and render it more susceptible to electrophilic oxidants such as the hydroxyl radical. Conversely, motifs that withdraw electron density from sulfur should render the residue more resistant to oxidation. Although a prior report indicates that methionine residues engaged in *S*–aryl interactions are, in general, less prone to oxidation, we note that neither the angle of approach nor the sulfur orientation was considered in that work [40]; we believe that structural nuances enable the *S*–aryl interaction to be either activating or deactivating with respect to oxidation. On this basis, we hypothesized that Met29 and Met35 should be more easily oxidized than Met13 or Met30. This hypothesis is supported by Baynes and coworkers, who determined that the rates of methionine oxidation in bovine RNase A are Met29 > Met30 > Met13 (Met79 was not oxidized, and RNase A lacks Met35) [41]. Bovine RNase A was not part of our homolog set, but investigation of its structure reveals that it possesses homologous *S*–aryl motifs (Figure S6) that conserve the interatomic distances, approach angles, and sulfur orientations observed in our homolog set. Taken together, our bioinformatics analyses suggested that in human RNase 1, Met29 and Met35 serve as sacrificial antioxidative residues intended to protect the active-site residues from oxidation.

### 2.3 | Capping Methionine with Ox2

We devised a chemical strategy to test the antioxidative role of methionine, analogous to protecting-group chemistry in organic synthesis. We reasoned that covalent modification of the methionyl sulfur atom would block its oxidation by ROS, effectively “capping” the residue and removing it from the antioxidative pool. To ensure that the only methionine residues present are those native to the mature protein (and exclude the N-terminal translation initiator methionine), we expressed RNase 1 as a fusion bearing an N-terminal enterokinase-cleavable tag, removed the tag proteolytically, and purified the authentic enzyme by chromatography. Mass spectrometry confirmed the expected molecular weight (Figure S7).

We reacted RNase 1 with the methionine-selective chemical probe Ox2 developed by Toste, Chang, and coworkers [42]. Ox2 is a bench-stable oxaziridine that undergoes chemoselective nitrogen-group transfer to the nucleophilic methionyl thioether. Importantly, the sulfimide product is resistant to further oxidation by ROS, ensuring that the capped methionine cannot act as a sacrificial antioxidant. We observed two predominant Ox2-labeling events (Figures 3A and S8). Of the five methionine residues in RNase 1, tryptic digestion of Ox2-labeled protein clearly identifies Met35 and eliminates Met79 as solvent-accessible targets, whereas a single labeling event was detected on the peptide harboring Met13, Met29, and Met30 (Figure S9). Heat denaturation experiments showed that the thermostability of RNase 1 is minimally destabilized by Ox2 labeling (Δ*T*_m_ = −4.0 ± 0.1°C; Figure S10). As measured with the fluorogenic substrate 6-FAM–dArU(dA)_2_–6-TAMRA (Figure S11) [43], the value of *k*_cat_/*K*_M_ for Ox2-labeled RNase 1 is (7.37 ± 0.06) × 10^5^ M^−1^ s^−1^, whereas that for unlabeled RNase 1 is (3.64 ± 0.06) × 10^6^ M^−1^ s^−1^. Thus, labeling RNase 1 with Ox2 perturbs enzymic stability and activity to only a minor extent, validating the use of Ox2 as a tool to interrogate the ability of methionine to act as a sacrificial antioxidant.

### 2.4 | Replacing Methionine with Norleucine

To complement the capping approach with a more conservative perturbation, we sought to replace Met29 and Met35 with an isosteric residue that lacks the redox-active sulfur atom. Considering that Ox2-labeling of RNase 1 methionines introduces steric bulk that could possibly confound the results of our subsequent oxidation-induced inactivation assay (e.g., by altering the conformation of the RNase 1 active site), we sought to install an amino acid that would remove the methionyl sulfur atom but not otherwise alter the residue side chain. Although no canonical amino acid meets these criteria, the noncanonical amino acid norleucine (Nle) is an isostere of methionine in which the thioether sulfur (─S─) is replaced with a methylene group (─CH_2_─), preserving side-chain length and hydrophobicity while eliminating redox activity. Moreover, substitution of norleucine for methionine has been used by others to interrogate the function of methionine in other proteins [44–46]. Because the site-selective installation of norleucine at positions 29 and 35 is not achievable by conventional recombinant expression (which would incorporate norleucine globally in place of all methionine residues [44–46]), we turned to total chemical synthesis.

The chemical synthesis of a ribonuclease has a storied history. In 1969, Gutte and Merrifield reported the total synthesis of bovine RNase A (124 residues) by solid-phase peptide synthesis, marking the first chemical synthesis of an enzyme [47, 48]. That landmark achievement, which contributed to Merrifield’s 1984 Nobel Prize in Chemistry [49], required 369 (= 3 × 123) chemical reactions and 11,931 automated steps performed over weeks [50]. Here, using automated fast-flow peptide synthesis (AFPS) [51], we assembled the entire 128-residue polypeptide chain of Met29Nle Met35Nle RNase 1 (“[Nle]RNase 1”) in 256 automated steps (coupling and deprotection of each residue) over <6 h with norleucine installed at the two predetermined positions, a level of synthetic expediency and control not accessible with other approaches.

We purified and folded the polypeptide, purified the folded protein by chromatography, and confirmed that the protein mass was consistent with the expected molecular weight of folded [Nle]RNase 1 (Figure 3B). Our yield of folded, purified [Nle]RNase 1 was 24% relative to the starting mass of the purified polypeptide (Table S2), consistent with results of other proteins synthesized via AFPS [51, 52]. The ability to obtain correctly folded, catalytically active protein rapidly from a single synthetic run underscores the practicality of AFPS for structure–function studies of single-domain proteins. We also synthetically prepared wild-type RNase 1 by AFPS to verify the functional equivalence of the recombinantly expressed and synthetic enzymes, ensuring that the results observed for [Nle]RNase 1 would not be confounded by artifacts from the synthetic production of the enzyme. Enzymatic catalysis provides an extremely sensitive measure of native protein structure [53]. Synthetic wild-type RNase 1 and [Nle]RNase 1 are highly active catalysts, having *k*_cat_/*K*_M_ values of (1.58 ± 0.06) × 10^6^ M^−1^ s^−1^ and (9.55 ± 0.03) × 10^5^ M^−1^ s^−1^, respectively. Thus, the norleucine variant retains ~60% of the activity of its direct synthetic wild-type control. The expected mass upon folding and retention of high catalytic activity indicate that [Nle]RNase 1 adopts a proper fold and is suitable for examining the function of Met29 and Met35 in RNase 1.

### 2.5 | Oxidation-Induced Inactivation

To investigate whether methionine acts as a sacrificial antioxidant to protect the oxidation-prone catalytic residues His12 and His119, we challenged unlabeled wild-type RNase 1, Ox2-labeled RNase 1, and [Nle]RNase 1 in an oxidation assay. Each protein was subjected to Fenton oxidation (Equation 1) conditions: varying concentrations of H_2_O_2_ with catalytic Fe^2+^ (100:1 H_2_O_2_/FeSO_4_) at 37°C for 3 h. Under these conditions, Fe^2+^ reduces H_2_O_2_ to generate hydroxyl radical (HO⋅), which is a powerful and indiscriminate oxidant that can react with histidine to produce 2-oxohistidine [54, 55].


(1) $${\text{Fe}}^{2+}+{\text{H}}_{2}{\text{O}}_{2}\to {\text{Fe}}^{3+}+\text{HO}\cdot +{\text{HO}}^{-}$$

The use of Fe^2+^ is particularly important, as the oxidation of biomolecules by hydrogen peroxide is slow relative to the reaction of the hydroxyl radical with amino acids [56, 57]. Following oxidation, the proteins were assayed for ribonucleolytic activity using 6-FAM–dArU(dA)_2_–6-TAMRA [43]. We hypothesized that because Ox2-labeled RNase 1 contains chemically capped Met29 and Met35, the enzyme should be rendered less protected against inactivation by oxidation of catalytic residues and should therefore lose catalytic competency at a lower number of equivalents of hydrogen peroxide than is required to inactivate unlabeled wild-type RNase 1 (Figure 4A). We likewise hypothesized that [Nle]RNase 1, in which the sulfur atom of Met29 and Met35 has been obviated by residue substitution with norleucine, should also be more sensitive to inactivation via oxidation (Figure 4A). As shown in Figure 4B, Ox2-labeled RNase 1 is indeed more susceptible to oxidation-induced inactivation: IC_50_ = 111 ± 8 equiv, versus IC_50_ = 222 ± 18 equiv for unlabeled RNase 1. Similarly, [Nle]RNase 1 is more easily inactivated by oxidation: IC_50_ = 89 ± 17 equiv. The equivalents required for attenuation of ribonucleolytic activity are consistent (within error) for both Ox2-labeled and norleucine-substituted RNase 1. The approximately twofold decrease in IC_50_ upon perturbation of Met29 and Met35 is consistent with the removal of two competing substrates for the hydroxyl radical, thereby increasing the effective concentration of oxidant available to react with the catalytic histidines. We note that our assay measures the loss of catalytic function as a highly sensitive proxy for active-site oxidation. Because the hydroxyl radical is an indiscriminate oxidant, macroscopic Fenton oxidation generates a complex, heterogeneous mixture of trace modifications that notoriously confounds site-specific quantification by tandem mass spectrometry. Nevertheless, the oxidation of His12 and His119 to 2-oxohistidine is the most parsimonious explanation for the observed inactivation in our assay. This functional readout cuts through the chemical noise and is consistent with previous results on the susceptibility of these residues to oxidation [24] and their absolute contribution to catalysis [21, 22]. Thus, our chemical biology and biomimetic approaches support the hypothesis that Met29 and Met35 serve an antioxidative, protective role for RNase 1 catalysis.

### 2.6 | Methionine Utilization in the Proteome

The chemical logic uncovered in RNase 1 may extend to other proteins. Methionine is one of the rarest amino acids in human proteins, comprising only 2.2% of residues [58], a frequency inflated by its retention as the translation initiator on proteins for which methionine aminopeptidase cannot cleave the N-terminal methionine [59]. In contrast, human RNase 1 is highly enriched in this otherwise rare amino acid: its five methionine residues constitute 3.9% of the 128-residue sequence, nearly double the proteomic average. Histidine, meanwhile, is greatly overrepresented in enzymic active sites: 21% of enzymes use histidine in their catalytic function [1, 2]. This overrepresentation is especially consequential because oxidative damage to histidine residues is irreversible: no enzyme is known to reduce 2-oxohistidine back to histidine, and restoring function therefore requires degradation and replacement of the entire protein. In contrast, methionine sulfoxide can be reduced back to methionine by methionine sulfoxide reductases [27], making the oxidation of methionine a reversible and tolerable event. Methionine thus serves as a uniquely suitable sacrificial antioxidant: it intercepts oxidants that would irreversibly damage histidine, while itself sustaining only reparable modification.

To assess whether methionine-based protection of histidine is a general phenomenon, we surveyed additional human secretory proteins that rely on histidine residues for biological function. In PAF acetylhydrolase, which slightly overutilizes methionine (2.6%), we found three methionine residues within 14 Å of His351 of the catalytic Ser-His-Asp triad (Figure 5A) [60]. In trypsin 1, which underutilizes methionine (0.89%), two Met residues cluster near His57 of its catalytic triad (Figure 5B) [61]. These observations suggest that spatial proximity to active-site histidines, rather than global overrepresentation, may be the more informative criterion for identifying protective methionine residues. Calprotectin contains a slew of methionine residues (3.9%), several of which are proximal to histidines that coordinate first-row transition metals (Figure 5C) [62]. Covalent modification of these histidines abolishes metal binding [63], yet methionine-oxidized calprotectin retains both high-affinity metal binding and antimicrobial activity [64, 65], consistent with methionine absorbing oxidative damage without functional cost. Because calprotectin is abundant in neutrophils, which generate ROS during the respiratory burst [66], such a mechanism may be especially valuable in this protein.

## 3 | Conclusion

In this work, we have combined bioinformatic analysis, chemoselective labeling, and total chemical synthesis to demonstrate that Met29 and Met35 of human RNase 1 function as sacrificial antioxidants that protect the catalytic histidines in the active site. Although this phenomenon was proposed three decades ago [25–28], it has not been verified experimentally, even in secretory ribonucleases, which are among the most studied proteins in chemistry. Bioinformatic analyses of human RNase 1 and 18 homologs revealed that methionine residues accumulate during the evolution of the RNase A superfamily, are frequently installed in the vicinity of the active site, and engage in *S*–aryl motifs with conserved aromatic residues that appear to tune their oxidative reactivity. The oxaziridine probe Ox2 is shown here to function as a chemical capping group for methionine sulfur, providing a general strategy for interrogating the redox function of specific methionine residues in native proteins. The total chemical synthesis of [Nle]RNase 1 via automated fast-flow peptide synthesis establishes that noncanonical amino acids can be site-selectively installed in this protein. Both the Ox2-labeling and norleucine-substitution strategies converge on the same conclusion: perturbation of Met29 and Met35 renders RNase 1 more susceptible to oxidation-induced inactivation.

The patterns of methionine conservation observed in the RNase A superfamily, together with our survey of other secretory proteins, suggest that the sacrificial deployment of methionine near catalytic or coordinating histidine residues is a conserved strategy. Because approximately 11% of the human proteome consists of soluble secretory proteins [67], and because 21% of enzymes utilize histidine in their catalytic function [2], the scope of methionine-based antioxidative protection may be substantial. Statistical overrepresentation of methionine within a protein sequence, or its spatial clustering near active-site histidines regardless of sequence frequency, may serve as a useful criterion for identifying additional proteins that employ this protective mechanism. More broadly, our findings reveal an underappreciated role for the thioether side chain of methionine: not merely as a hydrophobic building block, but as a kinetically accessible sacrificial reductant that absorbs oxidative damage, thereby preserving the chemical integrity of catalytically essential imidazole groups in oxidatively challenging environments.

## Supplementary Material

Supplementary Material

Additional supporting information can be found online in the Supporting Information section.

**SupportingFile**:anov70034-sup-0001-SuppMat.pdf.

## Figures and Tables

**FIGURE 1 |:**
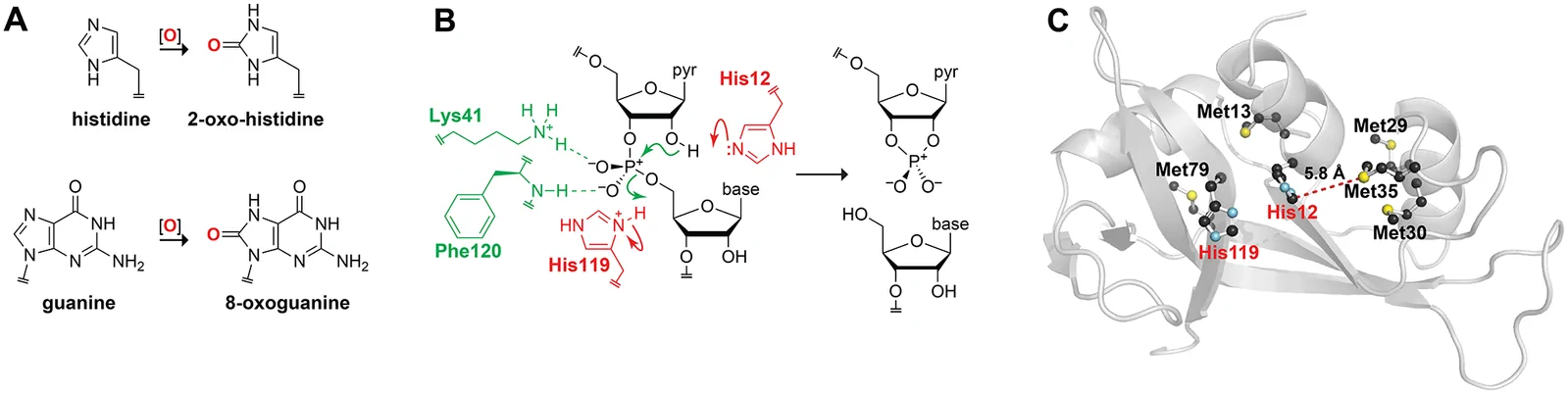
Chemical transformations. (A) Oxidation of histidine and guanine by reactive oxygen species (ROS). (B) Mechanism of catalysis by bovine RNase A and human RNase 1. His12 and His119 are shown in red; other important residues are shown in green. “pyr” refers to a pyrimidine nucleobase. (C) His12 and His119 in the active site of human RNase 1, surrounded by methionine residues (PDB entry 1Z7X).

**FIGURE 2 |:**
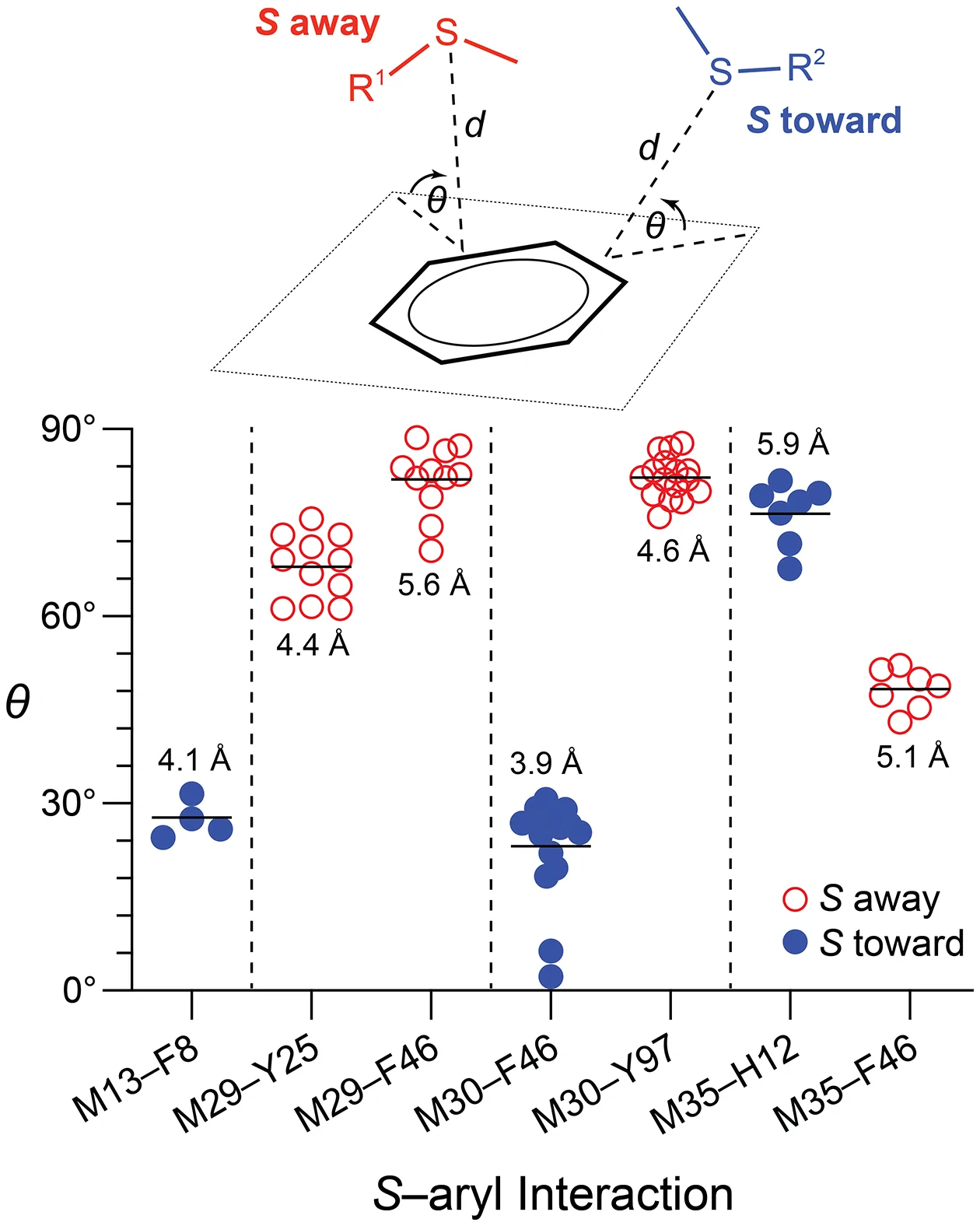
Graph of the methionyl angle of approach (*θ*), and orientation (“*S* away” or “S toward”) to aromatic residues for each *S*–aryl motif in RNase 1 homologs. The mean *S*–aryl distance (*d*) is indicated for each interaction. Residues are numbered according to their position in human RNase 1.

**FIGURE 3 |:**
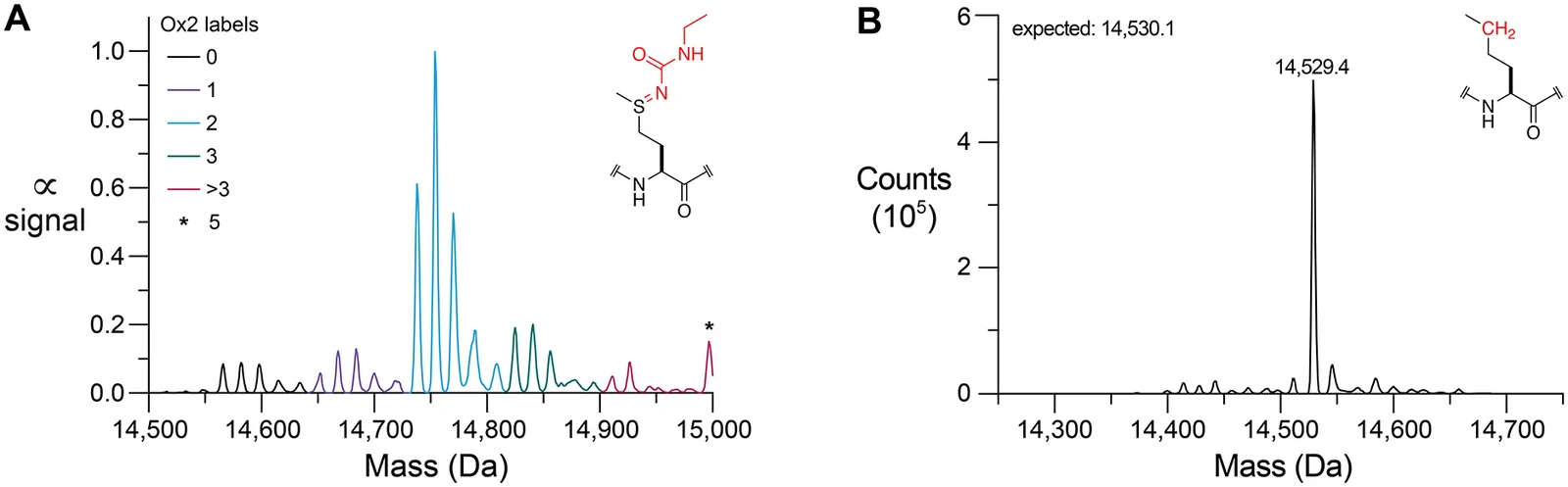
Deconvoluted Q–TOF mass spectra of oxidation-resistant variants of human RNase 1. (A) Ox2-labeled RNase 1; Δ*m*: +86.1 Da per Ox2 label. The predominant product contains two Ox2 labels. (B) [Nle]RNase 1; Δ*m*: −18.0 Da per Nle substitution. In each spectrum, closely spaced peaks correspond to adventitious oxidation events (Δ*m*: +16.0 Da per O addition).

**FIGURE 4 |:**
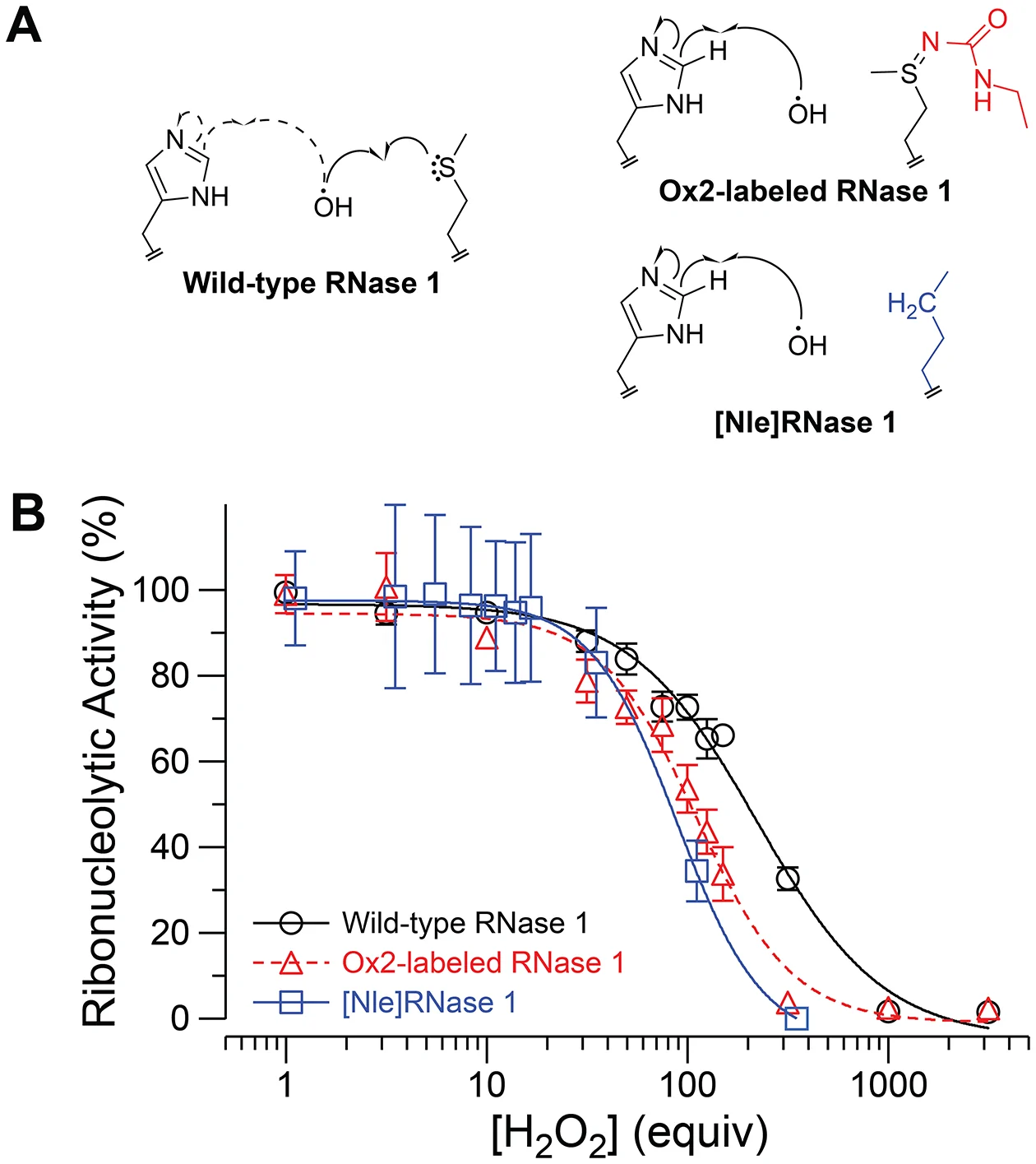
Oxidation-induced inactivation of RNase 1. (A) Proposed mechanism of attack of a ROS (here, hydroxyl radical) on wild-type RNase 1 and its variants. In each enzyme, an oxidant can react with the catalytic residues His12 or His119. In wild-type RNase 1, the oxidant can instead react with a proximal Met. This sacrificial antioxidative role of methionine is inaccessible in both Ox2-labeled RNase 1 and [Nle]RNase 1. (B) Semilog plot showing the oxidation-induced inactivation assay inhibition curves for wild-type, unlabeled RNase 1 (black line), Ox2-labeled RNase 1 (red dashed line), and [Nle]RNase 1 (blue line). Equivalents of hydrogen peroxide are rate-normalized to account for differing protein concentrations.

**FIGURE 5 |:**
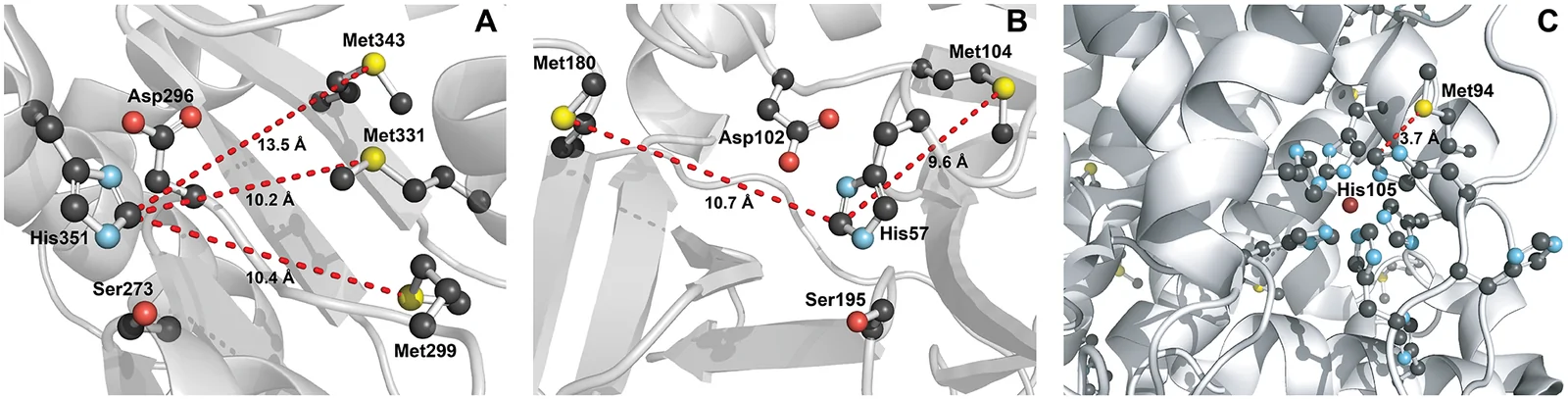
Methionine utilization in human secretory proteins with catalytic or coordinating histidines. Distances between proximal methionyl sulfur atoms and histidine carbons susceptible to oxidative attack are shown. (A) Environment surrounding the PAF acetylhydrolase catalytic triad (PDB entry 3D59 [60]). (B) Environment surrounding the trypsin 1 catalytic triad (PDB entry 1TRN [61]). (C) Environment surrounding a calprotectin metal-binding site of six histidine residues (PDB entry 5W1F [62]).

## Data Availability

The data that supports the findings of this study are available in the supplementary material of this article
