# Supplementary material for: Sacrificial Methionine Residues Protect Active-Site Histidines From Oxidation
Source: Angew Chem Novit. Author manuscript; Available in PMC 2026 Oct 26. (PMC13695568; doi:10.1002/anov.70034)
Supplement: Supplementary file 1 [file NIHMS2214668-supplement-Supplementary_Material.pdf]

# Sacrificial Methionine Residues Protect Active-Site Histidines from Oxidation

Evans C. Wralstad, Jessica Sayers, Giulio Fittolani, David Sarabia-Castillo, Alex J. Callahan, Bradley L. Pentelute & Ronald T. Raines*

Department of Chemistry, Massachusetts Institute of Technology, Cambridge, MA 02139-4307 (USA)
*E-mail: rtraines@mit.edu
Homepage: http://raineslab.com

## Table of Contents

**S1. Abbreviations ........ S2**
**S2. General Information and Instrumentation ........ S3**
**S3. Bioinformatic Methods ........ S5**
S3.1 Characterizing Methionine Conservation Patterns Among Homologs ........ S5
S3.2 Identifying *S*–Aryl Motifs Among Homologs ........ S5
**S4. Preparation of Recombinant RNase 1 ........ S5**
S4.1. Cloning and Expression of M–DDDDK–RNase 1 ........ S5
S4.2. Inclusion Body Isolation and Protein Folding ........ S6
S4.3. Purification of M–DDDDK–RNase 1 ........ S6
S4.4. Enterokinase Digestion and Purification of Recombinant RNase 1 ........ S7
**S5. Synthesis and Characterization of Ox2 ........ S7**
**S6. Labeling of RNase 1 with Ox2 ........ S7**
**S7. Tryptic Digestion of Ox2-Labeled RNase 1 ........ S8**
**S8. Differential Scanning Fluorimetry ........ S8**
**S9. Preparation of Synthetic [Nle]RNase 1 and Wild-Type RNase 1 ........ S8**
S9.1. Polypeptide Synthesis ........ S8
S9.2. Cleavage and Purification of Crude Polypeptides ........ S10
S9.3. Oxidative Folding and Purification of [Nle]RNase 1 and Wild-Type RNase 1 ........ S12
**S10. Mass Spectrometry ........ S13**
S10.1. Intact Protein Mass Spectrometry ........ S13
S10.2. Tryptic Peptide Protein Mass Spectrometry ........ S13
**S11. Oxidation-Induced Inactivation Assay ........ S14**
S11.1. Oxidation Conditions ........ S14
S11.2. Ribonucleolytic Activity Assay ........ S14
S11.3. Data Analysis ........ S16
**S12. Supplementary Figures S1–S11 ........ S17**
**S13. Supplementary Tables S1 and S2 ........ S28**
**S14. $^{1}$H NMR Spectrum of Ox2 ........ S30**
**S15. References ........ S31**

## S1. Abbreviations

| | |
|---|---|
| ACN | acetonitrile |
| AFPS | automated fast-flow peptide synthesis |
| ASA | fractional accessible surface area |
| BCA | bicinchoninic acid |
| CAMPO | conservation analysis of multiple protein alignments online |
| CPK | Corey–Pauling–Koltun (color scheme) |
| DEPC | diethyl pyrocarbonate |
| DMF | dimethylformamide |
| DMSO | dimethyl sulfoxide |
| DPBS | Dulbecco’s phosphate-buffered saline |
| DSF | differential scanning fluorimetry |
| DTT | dithiothreitol |
| EDTA | ethylenediaminetetraacetic acid |
| ESI | electrospray ionization |
| 6-FAM | 6-carboxyfluorescein |
| FPLC | fast protein liquid chromatography |
| FRET | Förster resonance energy transfer |
| HPLC | high-performance liquid chromatography |
| IAM | iodoacetamide |
| $IC_{50}$ | half-maximal inhibitory concentration |
| IPTG | isopropyl β-D-1-thiogalactopyranoside |
| $k_{cat}$ | catalytic rate constant (turnover number) |
| $K_M$ | Michaelis constant |
| LB | Luria–Bertani (medium) |
| LC-MS | liquid chromatography–mass spectrometry |
| MALDI-TOF | matrix-assisted laser desorption/ionization–time of flight |
| mCPBA | *meta*-chloroperoxybenzoic acid |
| Met | methionine |
| MetO | methionine sulfoxide |
| MUSCLE | multiple sequence comparison by log-expectation |
| Nle | L-norleucine |
| [Nle]RNase 1 | Met29Nle/Met35Nle RNase 1 |
| NMR | nuclear magnetic resonance |
| $OD_{600\ nm}$ | optical density at 600 nm |
| Ox2 | *N*-ethyl-3-phenyl-1,2-oxaziridine-2-carboxamide |
| PAF | platelet-activating factor |
| PDB | Protein Data Bank |
| Q-TOF | quadrupole time-of-flight |
| RMSD | root-mean-square deviation |
| RNase 1 | ribonuclease 1 (EC 4.6.1.18; *Homo sapiens*: UniProtKB P07998) |
| RNase A | ribonuclease A (EC 4.6.1.18; *Bos taurus*: UniProtKB P61823) |
| ROS | reactive oxygen species |
| SASA | solvent-accessible surface area |
| SD | standard deviation |

| | |
|---|---|
| SEM | standard error of the mean |
| 6-TAMRA | 6-carboxytetramethylrhodamine |
| TCEP | tris(2-carboxyethyl)phosphine |
| TFA | trifluoroacetic acid |
| $T_m$ | melting temperature (temperature at the midpoint of thermal denaturation) |
| Tris | tris(hydroxymethyl)aminomethane |
| UV | ultraviolet |
| VADAR | volume, area, dihedral angle reporter |

## S2. General Information and Instrumentation

All chemical reagents were purchased from commercial sources and used without further purification unless otherwise noted. Ultrapure water (≥18.2 MΩ cm) was used throughout. Dulbecco's phosphate-buffered saline (DPBS) was purchased from Gibco. All buffers used for ribonuclease work were treated with diethyl pyrocarbonate (DEPC) prior to use, except for Tris-containing buffers, which were prepared from RNase-free stocks due to the reactivity of the Tris amino group with DEPC.

**Conditions.** All procedures were performed in air at ambient temperature (~22 °C) and pressure (1.0 atm) unless indicated otherwise.

**Cloning and molecular biology.** The Q5 site-directed mutagenesis kit, NEB 10-beta *Escherichia coli* cells, bovine enterokinase light chain, and *Dpn*I restriction enzyme were obtained from New England BioLabs. Oligonucleotide primers were purchased from Integrated DNA Technologies. Plasmid purification was performed using the GeneJET Miniprep Kit (Thermo Fisher Scientific). Sanger sequencing was performed by Quintara Biosciences.

**Chromatography.** Fast protein liquid chromatography (FPLC) was performed on an ÄKTA system (Cytiva) using HiLoad 26/600 Superdex 75 prep grade and HiTrap Q columns (Cytiva).

Semi-preparative and preparative reversed-phase HPLC was performed using an Agilent 1260 Infinity II system equipped with a C4 column (Vydac, 10 × 250 mm, 5.0-µm particle, 300-Å pore) or a C3 column (Agilent Zorbax 300SB-C3, 9.4 × 250 mm, 5.0-µm particle, 300-Å pore). Fractions were manually collected based on UV absorption at 218 and 280 nm.

Analytical HPLC was performed using an Agilent 1290 Infinity II system equipped with a UV detector monitoring at 214 nm.

LC-MS total ion count (TIC) chromatograms and associated high-resolution mass-to-charge ($m/z$) spectra were acquired using an Agilent 6550 iFunnel Q-TOF LC-MS system. Analyses were performed on an Agilent 1290 Infinity HPLC coupled to an Agilent 6550 Q-TOF equipped with Dual Jet Stream electrospray ionization and iFunnel technology. Mass spectrometry data were acquired in positive ionization mode using extended dynamic range (2 GHz) over an $m/z$ range of 100–1,700. Data were processed using Agilent MassHunter Workstation Qualitative Analysis (version B.10.00) and Agilent MassHunter BioConfirm (version B.10.00).

Semi-preparative mass-directed reversed-phase high-performance liquid chromatography was performed using an Agilent mass-directed purification system consisting of a 1260 Infinity LC coupled to a 6130 Single Quadrupole MS and a Timberline Instruments TL105 column heater.

**Protein concentration.** Protein concentrations were determined by UV absorption at 215 nm ($\varepsilon$ = 227,448 $M^{-1}$ $cm^{-1}$) or by BCA assay (Thermo Fisher Scientific), as indicated. Centrifugal concentration was performed using Vivaspin 500 concentrators (Sartorius) or Amicon Ultra-15 concentrators (EMD Millipore).

**Mass spectrometry.** High-resolution electrospray ionization (ESI) mass spectrometry was performed on a 6530C Accurate-Mass Q-TOF mass spectrometer (Agilent Technologies) equipped with either a PLRP-S column (Agilent Technologies, 2.1 × 50 mm, 5-µm particle, 1000-Å pore) for intact proteins or a C18 column (Agilent Technologies, 3 × 150 mm, 2.7-µm particle, 120-Å pore) for tryptic peptides.

**Thermal denaturation.** Thermostability was determined by differential scanning fluorimetry (DSF), which was performed on a QuantStudio 7 Flex Real-Time PCR system (Applied Biosystems).

**Ribonucleolytic activity.** Fluorescence measurements were performed on a Tecan Spark multimode plate reader. The fluorogenic substrate 6-FAM–dArU$(dA)_2$–6-TAMRA was purchased from Microsynth AG.

**Polypeptide synthesis.** Automated fast-flow peptide synthesis (AFPS) was performed on the "Amidator" system described previously.[1] H-Rink amide ChemMatrix LL resin was used as the solid support.

**NMR spectroscopy.** $^1$H NMR spectra were acquired with a Bruker Avance-III HD Nanobay spectrometer operating at 400.09 MHz at the MIT Department of Chemistry Instrumentation Facility. Chemical shifts are reported in ppm relative to residual non-deuterated $CDCl_3$ ($\delta$ 7.26).

**Software.** Molecular visualization and distance measurements were made using PyMOL (Schrödinger). Phylogenetic analysis was performed using the Phylogeny.fr web server.[2] Sequence logos were generated using WebLogo version 3.7.4.[3] Protein structure analysis was performed using VADAR version 1.8.[4] Data were fitted using Prism version 10.0.3 (GraphPad). Thermal denaturation data were processed with Protein Thermal Shift software (Applied Biosystems).

## S3. Bioinformatic Methods

### S3.1. Characterizing Methionine Conservation Patterns Among Homologs

Human RNase 1 was queried by the Dali protein structure comparison server using PDB entry 1Z7X.[5,6] Preliminary query results were manually curated to remove entries that did not

correspond to an endogenous protein (*e.g.*, PDB-deposited structures of ribonucleases harboring heterologous mutations). Using the PyMod v3.0.2 plugin in a PyMOL environment, the curated homolog set ($n$ = 19 proteins) sequences were aligned using MUSCLE and structurally aligned using PyMOL.[7,8] For the distance between conserved methionine and catalytic residues, PyMOL was used to measure the distance from the methionyl sulfur atom to the histidine imidazole carbon atom between the two nitrogen atoms ($C^{\varepsilon 1}$), as this atom has been identified as an oxidative target.[9]

To classify methionine residues in the homolog set as solvent-exposed or buried, two methods were applied. In the first, PyMOL was used to measure the solvent-accessible surface area (SASA) of the methionyl sulfur atom; using the criterion set forth by Marino and Gladyshev, a methionine residue was considered exposed if the SASA of its sulfur atom was ≥0.1 Å$^2$.[10] In the second method, the VADAR model protein structure analysis server (v1.8) was used to measure the fractional accessible surface area (ASA) of all methionine residues.[4] Applying the criterion established by Aledo, Cantón, and Veredas, a methionine residue was characterized as exposed if its fractional ASA was ≥5%.[11] To harmonize these methods, a methionine residue was classified as exposed if at least one of these two criteria was met; otherwise, the residue was classified as buried.

Phylogenetic analysis of the homolog set was performed using the Phylogeny.fr web server with the previously generated MUSCLE sequence alignment.[2] Branch support was assessed using the approximate likelihood ratio test.

### S3.2. Identifying *S*–Aryl Motifs Among Homologs

A logo plot of the aligned homolog set sequences was generated using WebLogo v3.7.4.[3] The structures of the homolog set were colored by the sequence alignment using the CAMPO algorithm (Blosum62 scoring matrix; gap score: −1; gap-to-gap score: 0; gaps tossed).[12] *S*–Aryl motifs were identified using PyMOL to screen for distances ≤7 Å between conserved methionyl sulfur atoms and the nearest side-chain ring atom of aromatic residues. Approach angles were calculated using PyMOL and are defined as the angle between the plane of the aromatic ring and the line from the nearest ring atom to the methionyl sulfur atom. The sulfur orientation was defined based on which methionyl side-chain atoms were closest to the aromatic ring: "toward" if the methionyl sulfur atom was closest, "away" if the methionyl thioether carbon atoms were closest.

## S4. Preparation of Recombinant RNase 1

### S4.1. Cloning and Expression of M–DDDDK–RNase 1

The preparation of M–DDDDK–RNase 1 follows methods described previously.[13] Site-directed mutagenesis of a pET-22b(+)/human RNase 1 plasmid was conducted using the Q5 site-directed mutagenesis kit to install codons for Asp-Asp-Asp-Asp-Lys between the translation initiator Met codon and the gene sequence of mature RNase 1 using the following primers:

Forward: `5'-TGA TAA GAA AGA ATC TCG TGC TAA AAA ATT C-3'`
Reverse: `5'-TCA TCA TCC ATA TGT ATA TCT CCT TCT TAA AG-3'`

PCR amplification of the RNase 1 plasmid with the above primers was followed by 5′-phosphorylation of the linear amplicon, circularization by DNA ligase, and digestion of the template plasmid with *Dpn*I. NEB 10-beta *E. coli* cells were transformed by heat shock, grown overnight on 2% w/v LB–Miller agar plates containing ampicillin (200 μg $mL^{-1}$), and subsequently used to inoculate small-scale cultures (also LB–Miller media containing 200 μg $mL^{-1}$ ampicillin). The recombinant plasmid was purified by Miniprep; plasmids were verified by Sanger sequencing to contain the N-terminal enterokinase recognition tag.

Electrocompetent BL21(DE3) *E. coli* cells were transformed with the assembled plasmid by electroporation and selected on 200 μg $mL^{-1}$ ampicillin. LB-Miller containing ampicillin (200 μg $mL^{-1}$) and glucose (1% w/v) was inoculated with a single transformant and grown overnight at 37 °C with orbital shaking at 250 rpm. Bacteria were pelleted and resuspended in M9 medium (42.3 mM $Na_2HPO_4$, 22 mM $KH_2PO_4$, 18.7 mM $NH_4Cl$, 8.6 mM NaCl, 2 mM $MgSO_4$, 100 μM $CaCl_2$, 31 μM $FeCl_3$, 3.67 μM $ZnCl_2$, 0.74 μM $CuCl_2$, 0.42 μM $CoCl_2$, 81 nM $MnCl_2$, and 1.61 μM $H_3BO_3$) containing riboflavin, nicotinamide, pyridoxine hydrochloride, and thiamine (1 μg $mL^{-1}$ each); glucose (0.4% w/v); and ampicillin (200 μg $mL^{-1}$). Cultures were inoculated to an initial $OD_{600\ nm}$ of 0.05 and grown at 37 °C as before until $OD_{600\ nm}$ reached 1.00. Cultures were then supplemented with L-amino acids at the following concentrations: 100 mg $L^{-1}$ each lysine, threonine, and phenylalanine; 50 mg $L^{-1}$ each leucine, isoleucine, valine, and methionine. Protein overexpression was induced with IPTG (1 mM) for 4 h, with incubation as before. Cultures were pelleted by centrifugation, and pellets were stored at −70 °C.

### S4.2 Inclusion Body Isolation and Protein Folding

Pelleted *E. coli* cells were thawed and then resuspended in 20 mM Tris–HCl buffer, pH 7.6, containing EDTA (10 mM). Cells were passed through a cell disruptor (Constant Systems) at 22 kPSI, the lysate was subjected to centrifugation, and inclusion bodies were denatured in 20 mM Tris–HCl buffer, pH 8.0, containing guanidine–HCl (7 M), EDTA (10 mM), and DTT (100 mM). The solubilized protein was diluted tenfold with acetic acid (20 mM) and clarified by centrifugation; the supernatant was then dialyzed approximately 100-fold against acetic acid (20 mM) overnight at 4 °C to further clarify. Following centrifugation of the retentate, the supernatant was added dropwise to degassed folding buffer (which was 100 mM Tris–HCl buffer, pH 7.8, containing EDTA (10 mM), L-arginine (0.5 M), reduced glutathione (1.0 mM), and oxidized glutathione (0.2 mM)). The protein was allowed to fold at 4 °C without agitation for 5 days.

### S4.3. Purification of M–DDDDK–RNase 1

The folded protein was purified by gel-filtration (HiLoad 26/600 Superdex 75 prep grade, Cytiva) and cation-exchange (HiTrap Q, Cytiva) fast protein liquid chromatography; all chromatography buffers were treated with DEPC prior to use, except for Tris, which was added from ribonuclease-free stocks. The purified protein was buffer-exchanged into 20 mM Tris–HCl buffer, pH 7.2,

containing NaCl (50 mM). The protein was concentrated, quantified by BCA assay, flash-frozen in $N_2$(l), and stored at −70 °C.

### S4.4. Enterokinase Digestion and Purification of Recombinant RNase 1

M–DDDDK–RNase 1 was diluted to 1 mg mL$^{-1}$ using 20 mM Tris–HCl buffer, pH 7.2, containing NaCl (50 mM) and $CaCl_2$ (2 mM). Bovine enterokinase light chain (New England BioLabs) was added to the reaction mixture at 50 U enterokinase per 1 mg of M–DDDDK–RNase 1. The reaction mixture was gently agitated for 24 h. Completion of digestion with specific cleavage only at the N-terminal enterokinase recognition sequence was confirmed by mass spectrometry. Enterokinase-digested RNase 1 was buffer-exchanged into 50 mM sodium phosphate buffer, pH 2.7, and purified by semi-preparative reversed-phase HPLC on a C4 column (Vydac, 10 × 250 mm, 5.0-µm particle, 300-Å pore) that was equilibrated with the same buffer. The column was washed for 5 min with 12% v/v acetonitrile in 50 mM sodium phosphate buffer, pH 2.7, followed by a 12–60% v/v gradient (again with 50 mM sodium phosphate buffer, pH 2.7) for 50 min. Fractions were manually collected based on detection at $A_{218\text{ nm}}$ and $A_{280\text{ nm}}$. HPLC fractions confirmed by mass spectrometry to contain RNase 1 were combined in regenerated cellulose dialysis tubing (Spectrum Laboratories) and dialyzed 500-fold against 20 mM Tris–HCl buffer, pH 7.2, containing NaCl (50 mM). The protein was concentrated, quantified by BCA assay, flash-frozen in $N_2$(l), and stored at −70 °C.

## S5. Synthesis and Characterization of Ox2

The synthesis of the oxaziridine probe Ox2 followed the methods of Toste, Chang, and coworkers.[14] Briefly, benzaldehyde (12 mmol) and urea (10 mmol) were added to tetrahydrofuran (20 mL) along with titanium(IV) isopropoxide (14 mL). The reaction mixture was stirred gently for *ca*. 16 h; the crude mixture was then concentrated under reduced pressure, and the residue was dissolved in dichloromethane (30 mL). A solution of *m*-chloroperoxybenzoic acid (mCPBA, 30 mmol) was prepared in equal parts saturated potassium carbonate and dichloromethane (60 mL total); after stirring the mCPBA solution for 10 min, the crude product mixture was added dropwise to the mCPBA solution. The reaction mixture was stirred for a further 6 h, at which point water (100 mL) was added. The reaction mixture was extracted extensively with dichloromethane, and the organic layers containing Ox2 were combined. The product mixture was washed with brine, dried over $Na_2SO_4$(s), and concentrated under reduced pressure. The product was purified by column chromatography with 100:1 dichloromethane/diethyl ether.

**Characterization of Ox2.** $^1$H NMR (400 MHz, $CDCl_3$, $\delta$): 7.50–7.39 (m, 5H), 6.18 (s, 1H), 5.01 (s, 1H), 3.33 (m, 2H), 1.20 (t, $J$ = 7.3 Hz, 3H). The NMR data were consistent with those reported previously.[14]

## S6. Labeling of RNase 1 with Ox2

Recombinant RNase 1 was diluted to 1 mg mL$^{-1}$ protein (~69 μM) in 20 mM Tris–HCl buffer, pH 7.2, containing NaCl (50 mM). A stock solution of Ox2 was prepared in dimethylformamide at 350 mM; Ox2 (3.4 mM, 50 equiv) was then added. The reaction mixture was incubated for 30 min with gentle mixing on an orbital shaker. To remove excess Ox2 probe and terminate labeling, the reaction mixture was passed through a Zeba spin desalting column (Thermo Fisher Scientific) pre-equilibrated with DPBS (Gibco); the eluate was then passed through a second pre-equilibrated desalting column to ensure complete removal of the probe. Ox2-labeled RNase 1 was concentrated using a Vivaspin 500 centrifugal concentrator (Sartorius); protein concentration was quantified by UV absorption at 215 nm ($\varepsilon$ = 227,448 M$^{-1}$ cm$^{-1}$), and labeling was confirmed by mass spectrometry. Ox2-labeled RNase 1 was held at 4 °C and used within 24 h with verification of label retention.

## S7. Tryptic Digestion of Ox2-Labeled RNase 1

Tryptic peptides of Ox2-labeled RNase 1 were prepared using the Rapid-Digestion Trypsin kit from Promega. Digestion was conducted following the manufacturer's protocol with the following specifications: (1) 2 mM TCEP (Thermo Fisher Scientific) was used for sample reduction; (2) 5 mM iodoacetamide (Sigma–Aldrich) was used for sample alkylation; (3) RNase 1 was digested with Rapid Trypsin at a 10:1 protein/protease ratio with incubation at 70 °C for 45 min; (4) digestion was terminated by addition of trifluoroacetic acid (Sigma–Aldrich) to 0.5% v/v. Digests were desalted using C18 pipette tips (Thermo Fisher Scientific), flash-frozen in $N_2$(l), and lyophilized overnight.

## S8. Differential Scanning Fluorimetry

Differential scanning fluorimetry (DSF) was used to determine the thermostability of Ox2-labeled RNase 1 in relation to unlabeled RNase 1. (Note: Insufficient material precluded a determination of the thermostability of [Nle]RNase 1 by DSF.) SYPRO Orange protein gel stain (Supelco) was added to Ox2-labeled RNase 1 and unlabeled RNase 1 to achieve 1 mg mL$^{-1}$ protein and 50× SYPRO Orange (vendor stock: 5000× in DMSO) in DPBS (Gibco). The resulting samples were heated from 15–95 °C at 1 °C min$^{-1}$ using a QuantStudio 7 Flex Real-Time PCR system (Applied Biosystems); fluorescence was monitored in real-time with $\lambda_{ex}$ = 470 ± 15 nm and $\lambda_{em}$ = 586 ± 10 nm. Data were processed with Protein Thermal Shift software (Applied Biosystems) using the Boltzmann fitting method. Values of $T_m$ represent the temperature at which fluorescence reached 50% of its maximum.

## S9. Preparation of Synthetic [Nle]RNase 1 and Wild-Type RNase 1

### S9.1. Polypeptide Synthesis

The syntheses of Met29Nle/Met35Nle RNase 1 ([Nle]RNase 1) and wild-type RNase 1 were performed using the AFPS system with H-Rink amide ChemMatrix LL resin (142 mg, 0.17 mmol $g^{-1}$).[1] Solid-phase synthesis variables are summarized in the following table:

| Parameter | Condition(s) |
|---|---|
| Temperature | 85–90 °C in reactor, 60 °C in 5-min activation loop (for Cys and His), and 90 °C in 10-min activation loop (for all other amino acids) |
| Flow Rate | 40 mL $min^{-1}$ |
| Coupling Step | 0.40 M amino acid stock in amine-free DMF<br>0.38 M activator stock in amine-free DMF<br>Coupling conditions:<br>• HATU (13 pump strokes) for Asp, Glu, Gly, Ile, Leu, Lys, Met, Nle, Phe, Pro, Tyr<br>• HATU (26 strokes) for Ala and Ser<br>• PyAOP (26 strokes) for Arg, Asn, Cys, Gln, His, Thr, Val |
| Deprotection Step | 40% v/v piperidine in amine-free DMF containing 2% v/v formic acid (13 pump strokes) |
| Wash Step | Amine-free DMF (40 pump strokes) |

[Nle]RNase 1 has the amino acid sequence (with key residues highlighted):

```
  1   Lys-Glu-Ser-Arg-Ala-Lys-Lys-Phe-Gln-Arg
 11   Gln-His-Met-Asp-Ser-Asp-Ser-Ser-Pro-Ser
 21   Ser-Ser-Ser-Thr-Tyr-Cys-Asn-Gln-Nle-Met
 31   Arg-Arg-Arg-Asn-Nle-Thr-Gln-Gly-Arg-Cys
 41   Lys-Pro-Val-Asn-Thr-Phe-Val-His-Glu-Pro
 51   Leu-Val-Asp-Val-Gln-Asn-Val-Cys-Phe-Gln
 61   Glu-Lys-Val-Thr-Cys-Lys-Asn-Gly-Gln-Gly
 71   Asn-Cys-Tyr-Lys-Ser-Asn-Ser-Ser-Met-His
 81   Ile-Thr-Asp-Cys-Arg-Leu-Thr-Asn-Gly-Ser
 91   Arg-Tyr-Pro-Asn-Cys-Ala-Tyr-Arg-Thr-Ser
101   Pro-Lys-Glu-Arg-His-Ile-Ile-Val-Ala-Cys
111   Glu-Gly-Ser-Pro-Tyr-Val-Pro-Val-His-Phe
121   Asp-Ala-Ser-Val-Glu-Asp-Ser-Thr
```

Wild-type RNase 1 has the amino acid sequence (with key residues highlighted):

```
  1   Lys-Glu-Ser-Arg-Ala-Lys-Lys-Phe-Gln-Arg
 11   Gln-His-Met-Asp-Ser-Asp-Ser-Ser-Pro-Ser
 21   Ser-Ser-Ser-Thr-Tyr-Cys-Asn-Gln-Met-Met
 31   Arg-Arg-Arg-Asn-Met-Thr-Gln-Gly-Arg-Cys
 41   Lys-Pro-Val-Asn-Thr-Phe-Val-His-Glu-Pro
 51   Leu-Val-Asp-Val-Gln-Asn-Val-Cys-Phe-Gln
 61   Glu-Lys-Val-Thr-Cys-Lys-Asn-Gly-Gln-Gly
 71   Asn-Cys-Tyr-Lys-Ser-Asn-Ser-Ser-Met-His
 81   Ile-Thr-Asp-Cys-Arg-Leu-Thr-Asn-Gly-Ser
 91   Arg-Tyr-Pro-Asn-Cys-Ala-Tyr-Arg-Thr-Ser
101   Pro-Lys-Glu-Arg-His-Ile-Ile-Val-Ala-Cys
111   Glu-Gly-Ser-Pro-Tyr-Val-Pro-Val-His-Phe
121   Asp-Ala-Ser-Val-Glu-Asp-Ser-Thr
```

### S9.2. Cleavage and Purification of Crude Polypeptides

After synthesis, the resin was washed with dichloromethane and dried under $N_2$(g). The polypeptide was cleaved using 0.8 mL per µmol of a freshly prepared reducing cleavage mixture (77.5:5:5:5:5:2.5 trifluoroacetic acid/thioanisole:triisopropylsilane/trimethylsilyl chloride/dimethyl sulfide/ethanedithiol containing 0.1% w/v triphenylphosphine) with incubation for 3.5 h.[15] The cleavage mixture was drawn up with the reaction syringe containing the peptidyl resin, and the syringe was sealed with a Luer-tip syringe cap. The assembly was placed on a nutating mixer and incubated for 3.5 h; meanwhile, a 50-mL aliquot of diethyl ether was chilled on dry ice for >1 h. The cleavage supernatant in the reaction syringe was expelled into a 50-mL conical tube, and the resin was retained by the internal frit of the reaction syringe. An additional 2.5 mL of trifluoroacetic acid was drawn into the reaction syringe, and the contents were gently mixed; the aliquot was then expelled into the conical tube containing the cleavage supernatant. The pre-chilled diethyl ether was added to the cleavage supernatant, resulting in a white precipitate. The conical tube was subjected to centrifugation at 4000*g* for 10 min at 4 °C. The supernatant was removed, and the solid pellet was briefly dried under a stream of $N_2$(g). The dried peptide was dissolved in 5 mL of 1:1 water/acetonitrile containing trifluoroacetic acid (0.1% v/v) with vortexing and sonication as needed. The mixture was flash-frozen in $N_2$(l) and lyophilized. Yield of crude [Nle]RNase 1 polypeptide: 164 mg; yield of crude wild-type RNase 1 polypeptide: 107 mg.

The resulting crude material was dissolved in 100 mM Tris–HCl buffer, pH 7.0, containing guanidine–HCl (6 M). The crude material was purified by preparative reversed-phase HPLC (Agilent Zorbax 300SB-C3, 9.4 × 250 mm, 5.0-µm particle size, 300-Å pore) thermostatted at 50 °C. The column was washed for 10 min with 5% v/v acetonitrile in water containing trifluoroacetic acid (0.1% v/v), followed by a 5–20% v/v gradient for 15 min, and finally a 20–35% v/v gradient for 60 min. Fractions were collected in 20-s increments. HPLC fractions confirmed by mass spectrometry to be of high purity were combined and lyophilized to afford purified [Nle]RNase 1 polypeptide. Yield of purified [Nle]RNase 1 polypeptide: 22.8 mg; yield of purified wild-type RNase 1 polypeptide: 9.0 mg.

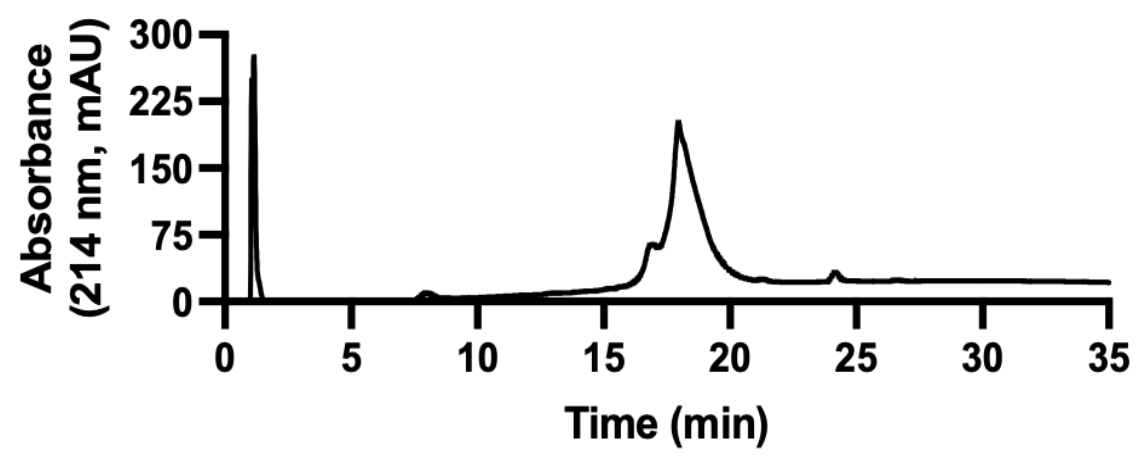


**Analytical HPLC of crude [Nle]RNase 1 polypeptide.** Column: Zorbax 300SB-C3 (150 × 2.1 mm, 5 µm); flow rate 0.4 mL/min; Solvent: A = $H_2O$ + 0.1% v/v TFA, B = ACN + 0.08% v/v TFA; Gradient: 5 min isocratic 5% B, 30 min linear gradient 5–65% B, 5 min isocratic 65% B.

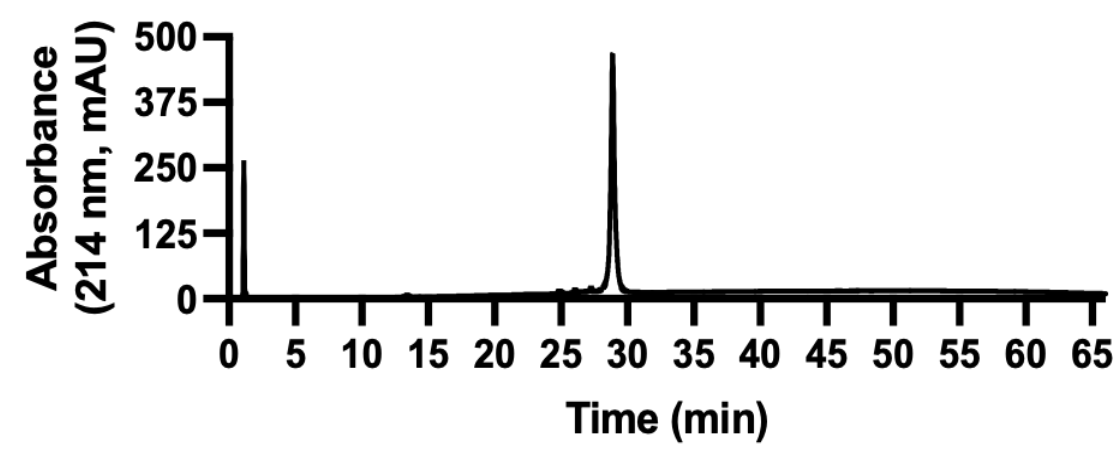


**Analytical HPLC of purified [Nle]RNase 1 polypeptide.** Column: Zorbax 300SB-C3 (150 × 2.1 mm, 5 µm); flow rate: 0.4 mL/min; Solvent: A = $H_2O$ + 0.1% v/v TFA, B = ACN + 0.08% v/v TFA; Gradient: 5 min isocratic 5% B, 30 min linear gradient 5–65% B, 5 min isocratic 65% B.

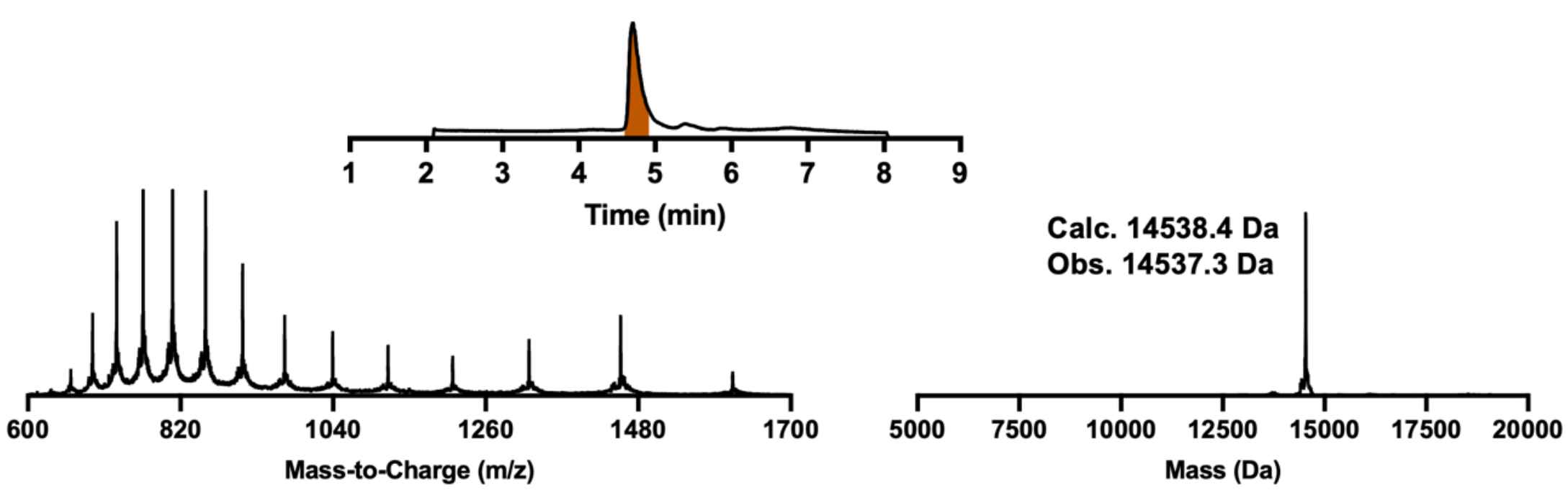


**LC-MS of purified [Nle]RNase 1 polypeptide.** TIC versus time, MS in positive ionization mode, extended dynamic range (2 GHz), and low mass range (*m*/*z* in range 100 to 1700). Column: Phenomenex Aeris C4 Wide Pore (5 µm, 150 × 2.1 mm, 300 Å silica); flow rate: 0.4 mL/min; A = $H_2O$ + 0.1% v/v formic acid, B = ACN + 0.1% v/v formic acid; Gradient: 0–2 min isocratic 1% B, 2–8 min linear gradient 1–91% B, 8–10 min linear gradient 91–95% B.

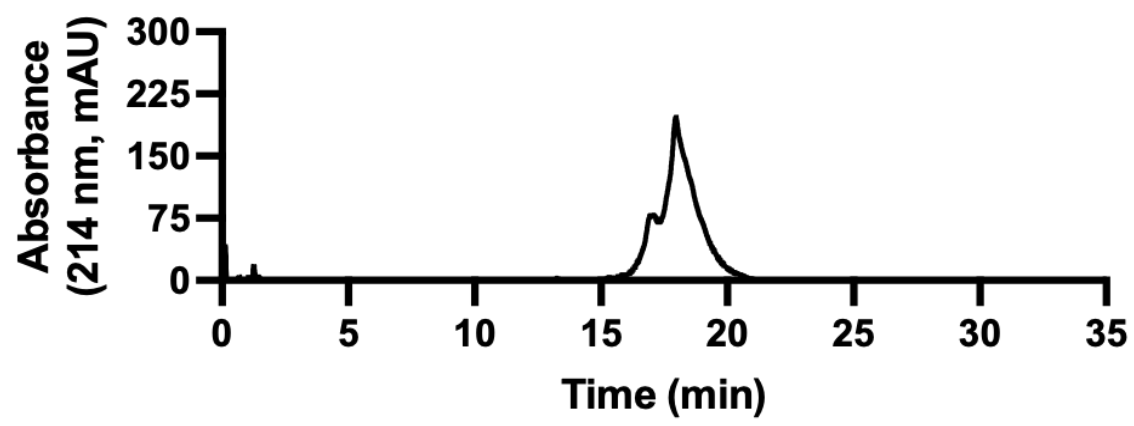


**Analytical HPLC of crude wild-type RNase 1 polypeptide.** Column: Zorbax 300SB-C3 (150 × 2.1 mm, 5 µm); flow rate 0.4 mL/min; Solvent: A = $H_2O$ + 0.1% v/v TFA, B = ACN + 0.08% v/v TFA; Gradient: 5 min isocratic 5% B, 30 min linear gradient 5–65% B, 5 min isocratic 65% B.

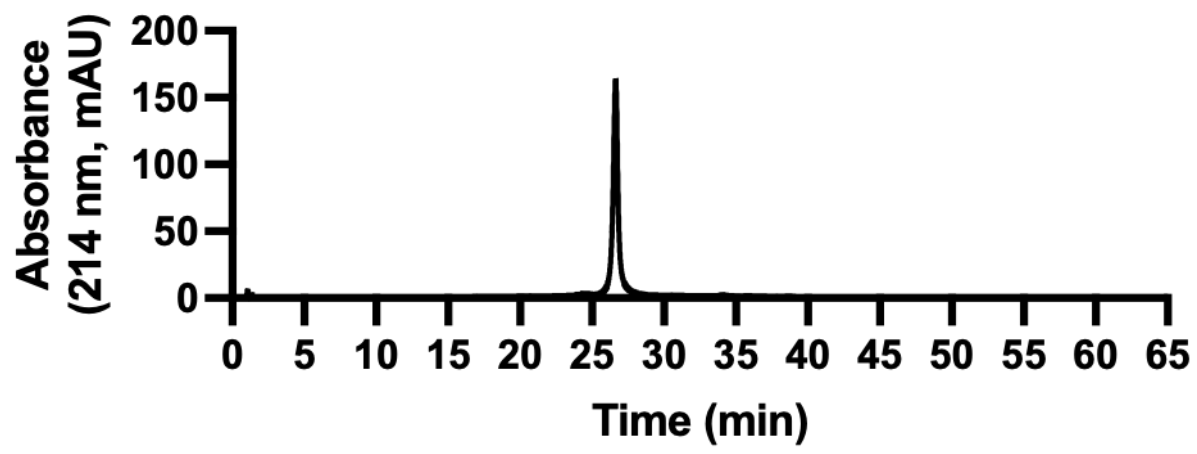


**Analytical HPLC of purified wild-type RNase 1 polypeptide.** Column: Zorbax 300SB-C3 (150 × 2.1 mm, 5 µm); flow rate: 0.4 mL/min; Solvent: A = $H_2O$ + 0.1% v/v TFA, B = ACN + 0.08% v/v TFA; Gradient: 5 min isocratic 5% B, 60 min linear gradient 5–65% B, 5 min isocratic 65% B.

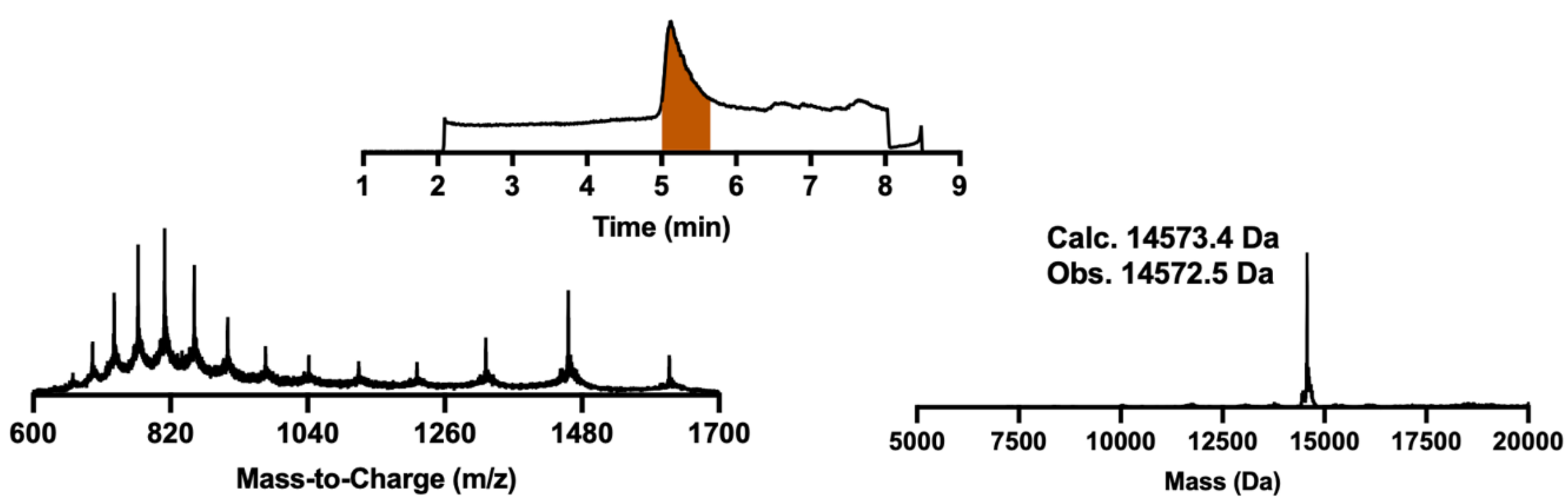


**LC-MS of purified wild-type RNase 1 polypeptide.** TIC versus time, MS in positive ionization mode, extended dynamic range (2 GHz), and low mass range (*m*/*z* in range 100 to 1700). Column: Phenomenex Aeris C4 Wide Pore (5 µm, 150 × 2.1 mm, 300 Å silica); flow rate: 0.4 mL/min; A = $H_2O$ + 0.1% v/v formic acid, B = ACN + 0.1% v/v formic acid; Gradient: 0–2 min isocratic 1% B, 2–8 min linear gradient 1–91% B, 8–10 min linear gradient 91–95% B.

### S9.3. Oxidative Folding and Purification of [Nle]RNase 1 and Wild-Type RNase 1

Each purified polypeptide was dissolved at 2 mg $mL^{-1}$ in 20 mM acetic acid containing EDTA (10 mM); the mixture was gently agitated at 37 °C for 30 min to ensure full dissolution of the

polypeptide. The polypeptide solution was analyzed by mass spectrometry to confirm a molecular weight consistent with eight reduced cysteine residues. (Folded RNase 1 has four disulfide bonds.) The polypeptide solution was added dropwise to degassed folding buffer (vide supra) while stirring gently at 4 °C, affording a final polypeptide concentration of ~50 μg $mL^{-1}$. After the polypeptide solution was fully added to the folding buffer, the mixture was incubated at 4 °C without agitation for 5 days.

Following protein folding, the protein solution was buffer-exchanged into 50 mM sodium phosphate buffer, pH 2.7, and was concentrated to 3 mg $mL^{-1}$ using an Amicon Ultra-15 centrifugal concentrator (EMD Millipore). The buffer-exchanged protein solution was passed through a Spin-X centrifugal tube filter (0.22-μm, cellulose acetate membrane; R&D Systems). The folded protein was then purified by preparative reversed-phase HPLC using a C4 column (Vydac, 10 × 250 mm, 5.0-μm particle, 300-Å pore) that was equilibrated with 50 mM sodium phosphate buffer, pH 2.7. The column was washed for 5 min with 12% v/v acetonitrile in 50 mM sodium phosphate buffer, pH 2.7, followed by a 12–36% v/v gradient (again with 50 mM sodium phosphate buffer, pH 2.7) for 60 min. Fractions were manually collected based on UV absorption at 218 nm and 280 nm. HPLC fractions confirmed by mass spectrometry to contain protein were placed under a gentle stream of $N_2$(g) overnight to reduce sample volume and acetonitrile content. The protein solution was adjusted to pH 5 using dibasic sodium phosphate, loaded into regenerated cellulose dialysis tubing (Spectrum Laboratories), and dialyzed 500-fold against 20 mM Tris–HCl buffer, pH 7.2, containing NaCl (50 mM). The protein was concentrated, quantified by UV absorption at 215 nm ($\varepsilon$ = 227,448 $M^{-1}$ $cm^{-1}$), flash-frozen in $N_2$(l), and stored at −70 °C.

## S10. Mass Spectrometry

### S10.1. Intact Protein Mass Spectrometry

High-resolution mass spectrometry of ribonucleases in solution was performed using ESI mass spectrometry on a 6530C Accurate-Mass Q-TOF mass spectrometer equipped with a PLRP-S column (Agilent Technologies, 2.1 × 50 mm, 5-μm particle, 1000-Å pore). A gradient of 5–95% v/v acetonitrile (0.1% v/v formic acid) in water (0.1% v/v formic acid) over 7 min was used. Before Q-TOF LC-MS analysis, all proteins were diluted to 2 μM in 0.1% v/v formic acid and passed through a Spin-X centrifugal tube filter (0.22-μm, cellulose acetate membrane; R&D Systems). Deconvolution of multiply charged ion envelopes was performed using MassHunter BioConfirm software (Agilent Technologies).

### S10.2. Tryptic Peptide Mass Spectrometry

For high-resolution mass spectrometry of tryptic peptides, lyophilized peptides were redissolved in ultrapure water containing formic acid (0.1% v/v) to ~5 μM (based on the starting protein concentration). The peptide solution was passed through a Spin-X centrifugal tube filter as described above for intact protein mass spectrometry. Samples were analyzed by ESI mass spectrometry using a 6530C Accurate-Mass Q-TOF mass spectrometer equipped with a C18 column (Agilent Technologies, 3 × 150 mm, 2.7-μm particle, 120-Å pore) and a gradient of 5–

40% v/v acetonitrile (0.1% v/v formic acid) in water (0.1% v/v formic acid) over 15 min. Peptide identification was performed using a ≤10 ppm mass accuracy cut-off. The following variable modifications were considered during peptide assignment: cysteine alkylation with iodoacetamide (+57.02 Da, +IAM), methionine oxidation (+15.99 Da, +MetO), methionine labeling with Ox2 (+86.04 Da, +Ox2), asparagine/glutamine deamidation (+0.98 Da, $-NH_2$), and methionine dethiomethylation (−48.00 Da, $-SCH_4$). Sequence coverage was 90.6% (Figure S9).[16]

## S11. Oxidation-Induced Inactivation Assay

### S11.1. Oxidation Conditions

**Recombinant RNase 1 and Ox2-labeled RNase 1.** Protein (1 mg $mL^{-1}$) was mixed with 1–3160 equiv hydrogen peroxide (with respect to protein) in DPBS; iron(II) sulfate was included in all oxidation samples at a ratio of 100:1 hydrogen peroxide/iron(II) sulfate. Samples were incubated at 37 °C for 3 h to oxidize the protein. Bovine liver catalase (Sigma–Aldrich) was then added at 50 µg $mL^{-1}$, and samples were incubated at 37 °C for a further 10 min to degrade any remaining hydrogen peroxide and halt oxidation.

**[Nle]RNase 1.** Due to the limited yield of folded, purified protein, [Nle]RNase 1 samples were oxidized at 0.333 mg $mL^{-1}$ with 1–3160 equivalents of hydrogen peroxide (with respect to protein) in DPBS; the molar concentrations of hydrogen peroxide were therefore also threefold lower than their corresponding concentrations for recombinant RNase 1 and Ox2-labeled RNase 1. Iron(II) sulfate was included in all oxidation samples at a ratio of 100:1 hydrogen peroxide to iron(II) sulfate. [Nle]RNase 1 samples were otherwise oxidized under the same conditions as for recombinant RNase 1 and Ox2-labeled RNase 1. Catalase quenching was performed identically.

### S11.2. Ribonucleolytic Activity Assay

Ribonucleolytic activity was measured using the fluorogenic substrate 6-FAM–dArU(dA)$_2$–6-TAMRA, which possesses a single scissile phosphodiester bond (Figure S11).[17] Cleavage of this bond relieves FRET quenching and increases FAM fluorescence.

**Recombinant RNase 1.** Oxidized protein samples were diluted to 100 pM with RNase-free assay buffer (100 mM Tris–HCl buffer, pH 7.5, containing 100 mM NaCl). To render the assay buffer RNase-free, 100 mM NaCl was DEPC-treated before the addition of Tris (from RNase-free stocks), due to the reactivity of the Tris amino group with DEPC. Equal volumes of oxidized protein and substrate were mixed to afford 50 pM oxidized protein and 200 nM substrate in assay buffer. Using a Tecan Spark multimode plate reader thermostatted to 25 °C, substrate fluorescence was measured at $\lambda_{ex}$ = 493 ± 5 nm and $\lambda_{em}$ = 515 ± 5 nm before addition of oxidized protein to determine the background fluorescence, $I_0$. After adding protein, the reaction was incubated at 25 °C and continuously monitored for 4 min. Bovine RNase A was then added to a final concentration of ~10 µM to cleave all of the substrate and determine $I_{max}$.

**Ox2-labeled RNase 1.** Oxidized protein samples were assayed under the same conditions as those used for recombinant RNase 1, except oxidized samples were diluted to 400 pM and mixed with substrate to afford 200 pM oxidized protein and 200 nM substrate in assay buffer. This higher protein concentration was used to ensure an adequate rate of product generation over the 4-min monitoring period, as Ox2 labeling lowers the catalytic efficiency of RNase 1.

**Synthetic [Nle]RNase 1 and Wild-Type RNase 1.** Oxidized protein samples were assayed under the same conditions as those used for recombinant RNase 1 and Ox2-labeled RNase 1, except oxidized samples were diluted to 250 pM and mixed with fluorogenic substrate to afford 125 pM [Nle]RNase 1 or 50 pM wild-type RNase 1, and 200 nM substrate in assay buffer. The higher protein concentration of [Nle]RNase 1 was used to ensure an adequate rate of product generation over the 4-min monitoring period, as norleucine incorporation lowers the catalytic efficiency of RNase 1.

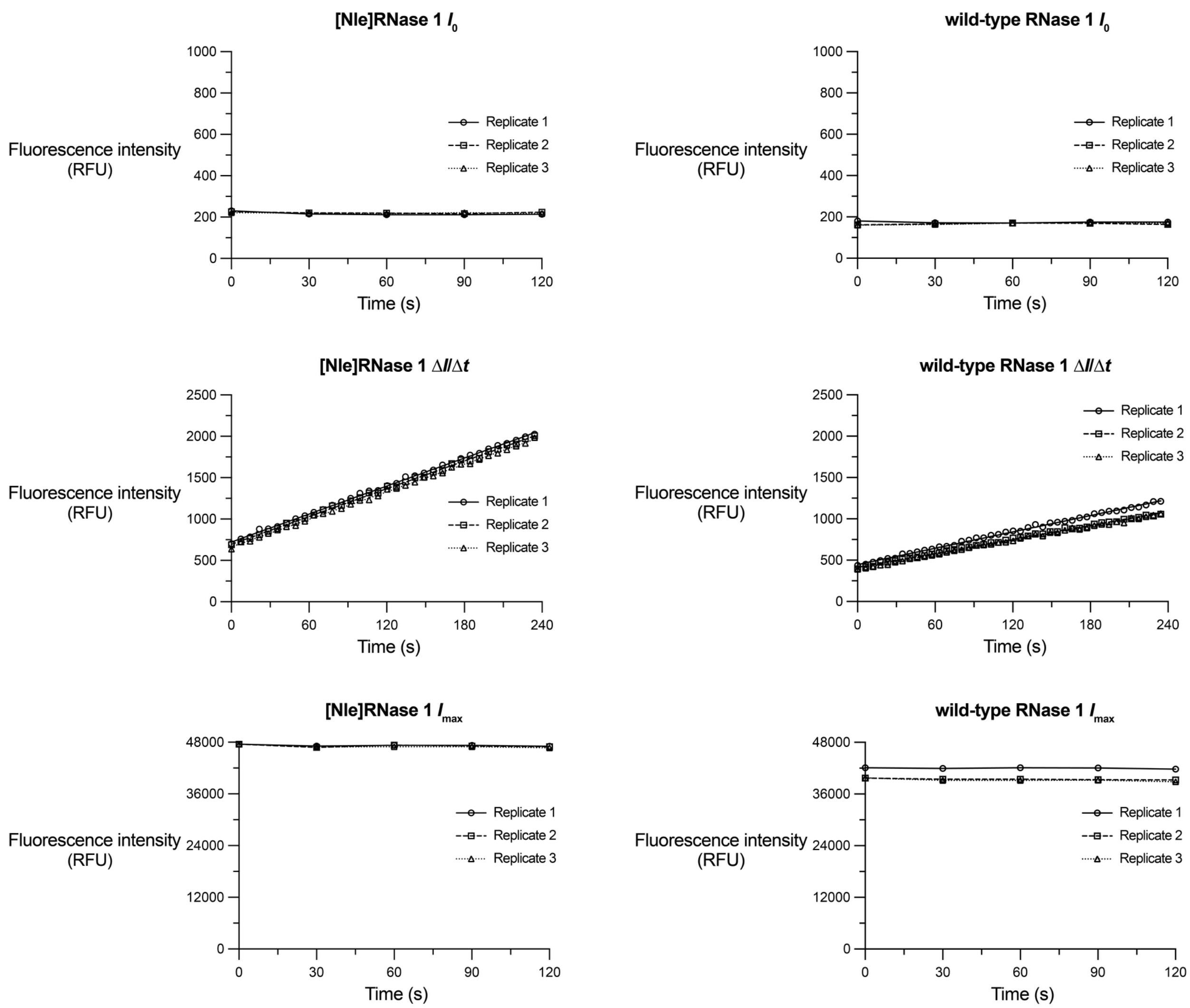

| Parameter | Synthetic [Nle]RNase 1 Replicate | | | Synthetic Wild-Type RNase 1 Replicate | | |
|---|---|---|---|---|---|---|
| | 1 | 2 | 3 | 1 | 2 | 3 |
| $\Delta I/\Delta t$ (RFU/s) | 5.641 | 5.592 | 5.550 | 3.513 | 2.977 | 2.988 |
| $I_0$ (RFU) | 216.6 | 221.4 | 219.4 | 174.6 | 166.2 | 168.0 |
| $I_{max}$ (RFU) | 47277 | 47162 | 47007 | 41965 | 39356 | 39166 |
| [E] (pM) | 125 | 125 | 125 | 50 | 50 | 50 |

### S11.3. Data Analysis

The value of $k_{cat}/K_M$ was calculated using the equation:

$$\frac{k_{cat}}{K_M} = \frac{\Delta I/\Delta t}{(I_{max} - I_0)[E]} \tag{S1}$$

where $\Delta I/\Delta t$ is the slope of the fluorescence versus time graph collected during the 4-min observation period and [E] is the concentration of enzyme.[17]

For each of the three proteins, the catalytic efficiency of each oxidized sample was normalized to the catalytic efficiency of an equivalent sample of the same protein that had not undergone oxidation. Plots of normalized reaction rates versus equivalents of hydrogen peroxide were fitted to a four-parameter logistic function using Prism version 10.0.3.

Because the concentrations of protein and hydrogen peroxide are each threefold lower for [Nle]RNase 1 than for the other two proteins, the rate of the bimolecular reaction of [Nle]RNase 1 with hydrogen peroxide is ninefold slower than the corresponding reaction for the other two proteins; to account for this difference, equivalents of hydrogen peroxide were normalized by the rate of the oxidation reaction. $IC_{50}$ values represent the number of equivalents of hydrogen peroxide required to reduce ribonucleolytic activity by 50%.

## S12. Supplementary Figures S1–S11

**A**

| Species | Protein | PDB ID | RMSD | % Identity |
|---|---|---|---|---|
| *M. musculus* | RNase 1 | 3tsra | 0.7 | 71 |
| *R. norvegicus* | RNase A | 1rraa | 0.7 | 66 |
| *S. scrofa* | RNase 4 | 5ar6a | 1.2 | 46 |
| *H. sapiens* | RNase 4 | 1rnfa | 1.2 | 44 |
| *C. mydas* | egg white RNase | 2zpoa | 1.4 | 42 |
| *B. taurus* | RNase 5 | 1agia | 1.7 | 34 |
| *R. norvegicus* | RNase 5 | 4qfib | 1.7 | 41 |
| *B. taurus* | seminal RNase | 11ba(A) | 0.6 | 72 |
| *M. musculus* | RNase 5 | 2bwla | 1.6 | 38 |
| *H. sapiens* | RNase 6 | 4x09a | 1.6 | 36 |
| *H. sapiens* | RNase 5 | 1a4yb | 1.7 | 38 |
| *H. sapiens* | RNase 3 | 1h1ha | 1.8 | 34 |
| *D. rerio* | RNase 5 | 3ljea | 1.7 | 35 |
| *G. gallus* | RNase 1 | 4perb | 2.1 | 34 |
| *H. sapiens* | RNase 7 | 2hkya | 2.1 | 39 |
| *L. pipiens* | amphinase | 2p6za | 1.9 | 29 |
| *L. catesbeianus* | RC-RNase 4 | 1kvza | 2.6 | 24 |
| *L. catesbeianus* | RC-RNase 2 | 1m58a | 2.2 | 27 |

**B**

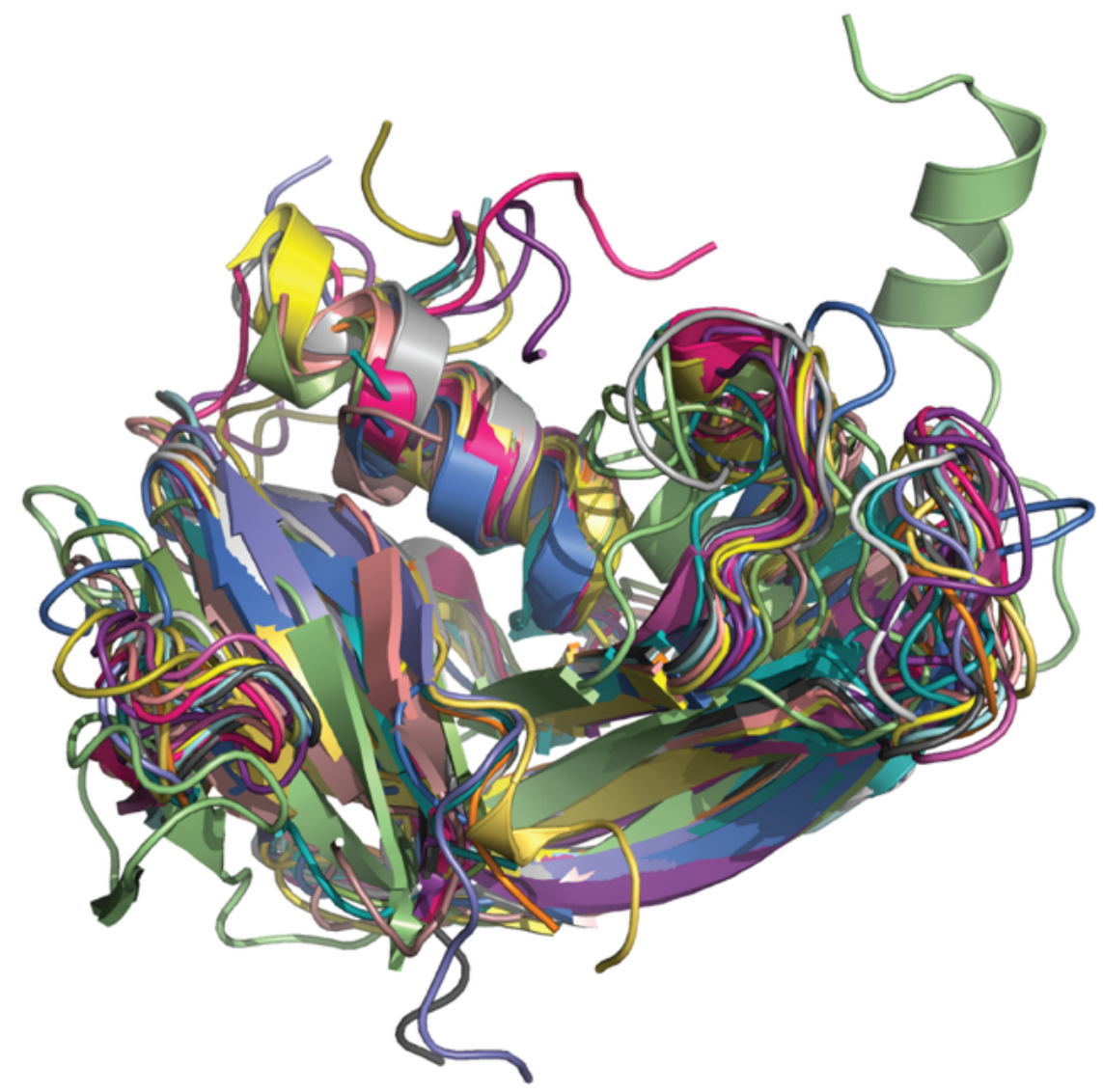

**Figure S1.** Homologs of human RNase 1. (A) 18 RNase 1 homologs identified by the Dali protein structure conservation server.[6] "RMSD" (in Å) refers to main-chain atoms. "% Identity" refers to the amino acid sequence identity with human RNase 1. (B) Structural alignment of the 19 members of the homolog set.

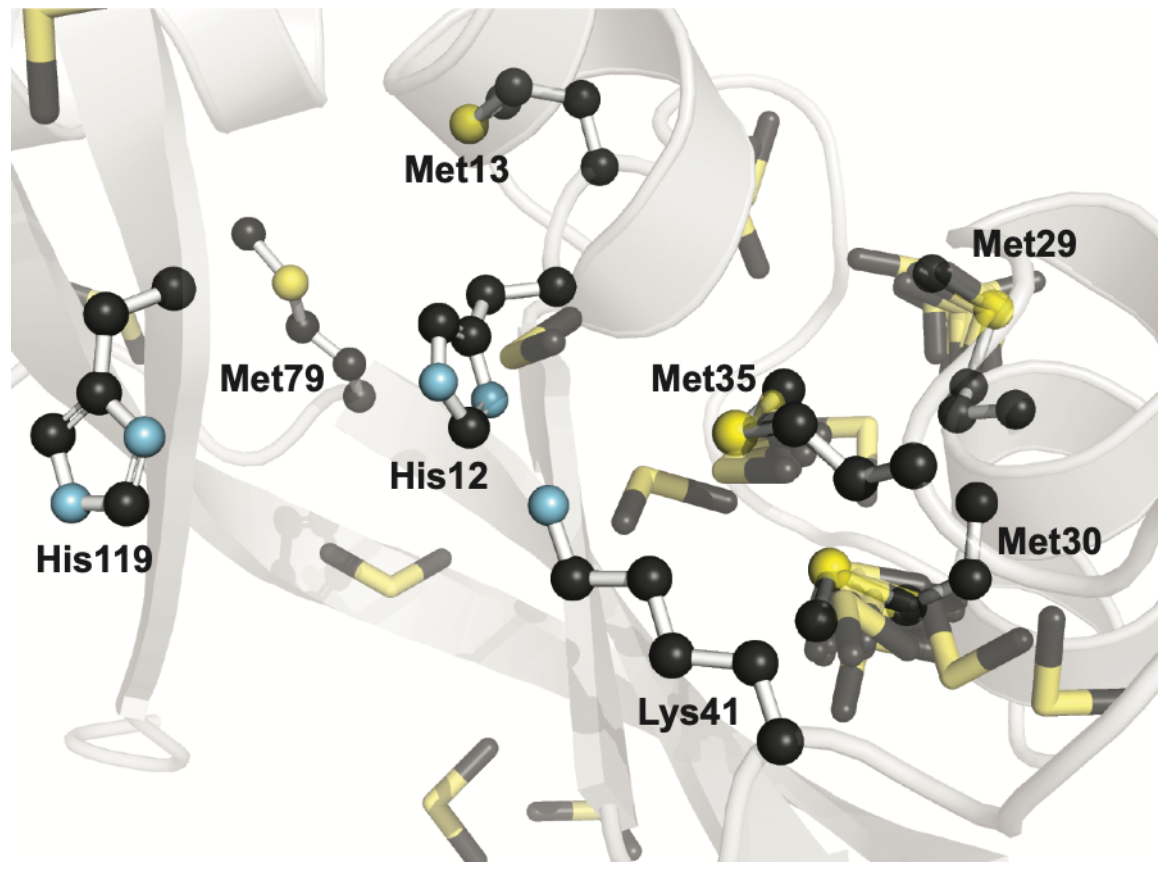


**Figure S2.** Conserved methionines of the homolog set relative to human RNase 1. The cartoon structure of RNase 1 is depicted with active-site residues and methionine side chains in ball-and-stick form (CPK color scheme); the methionine residues of the homologs are shown in stick form.

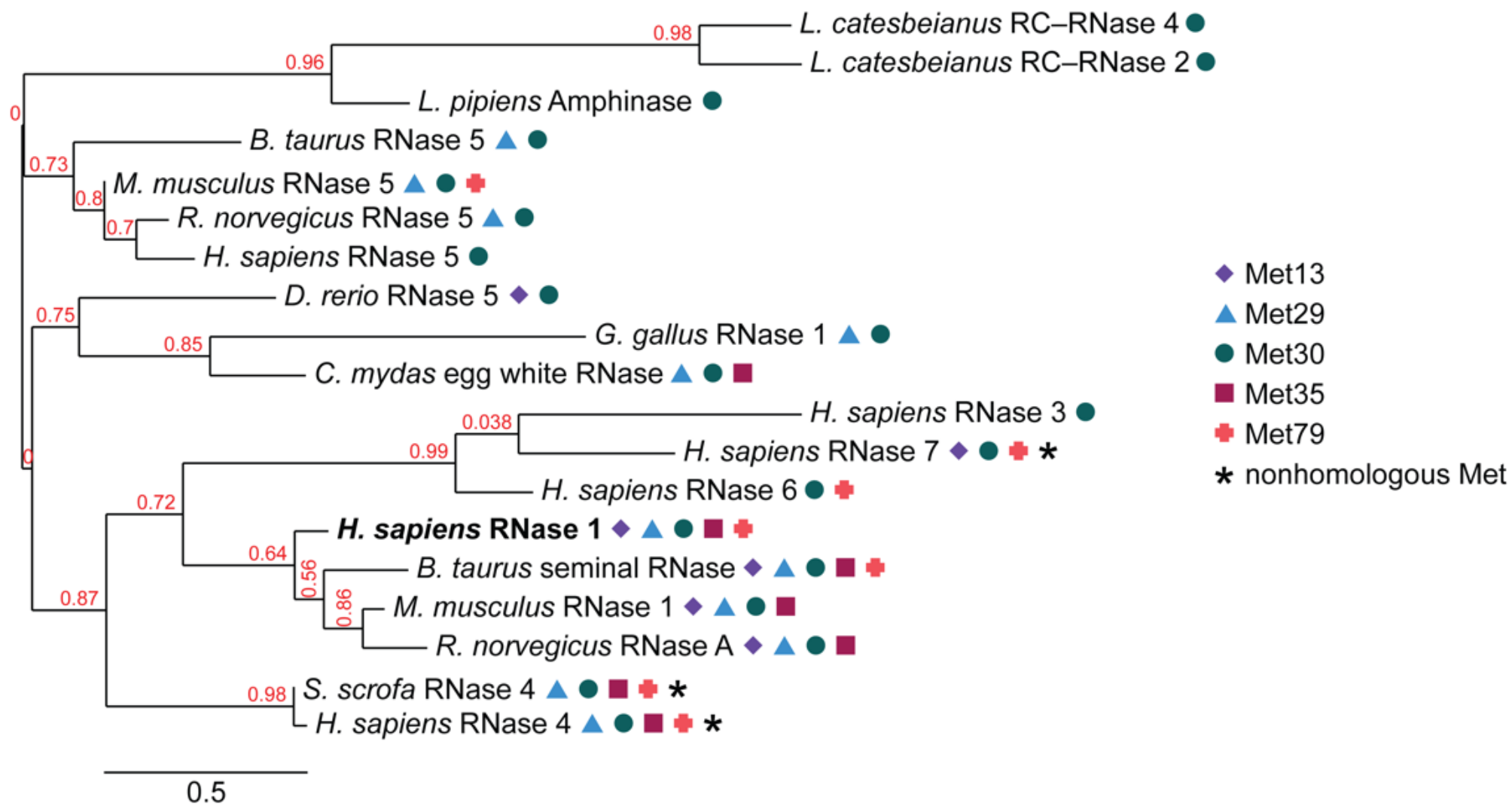


**Figure S3.** Phylogenetic tree of the human RNase 1 and 18 homologs. Symbols represent conserved methionine residues present in a homolog. Residues are numbered according to their equivalent position in human RNase 1. Node support is depicted in red.

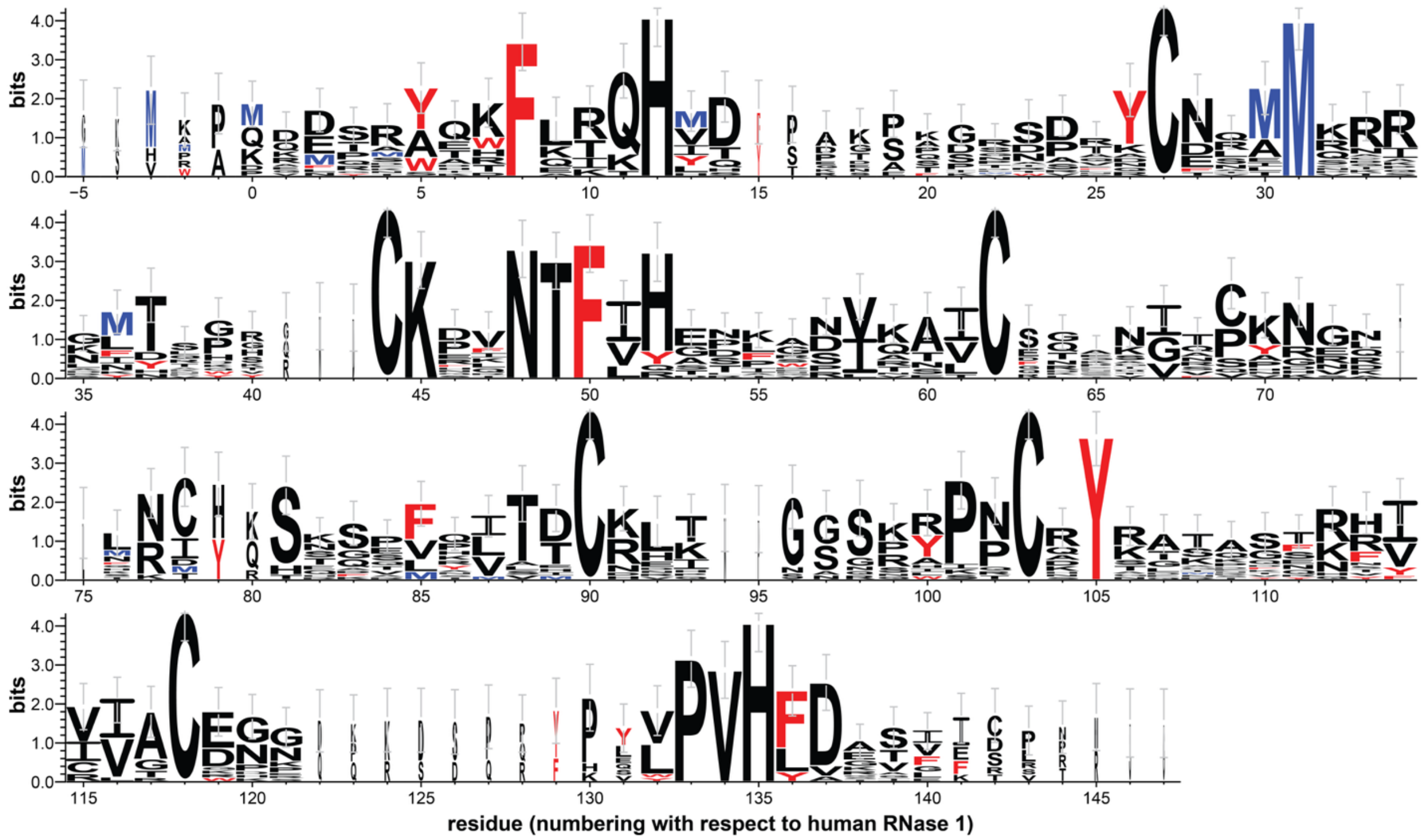


**Figure S4.** Sequence logo of human RNase 1 and 18 homologs. Residues are numbered with respect to the sequence of human RNase 1; numbering is approximate due to the presence of sequence gaps in the alignment of the homologs. Conserved methionine residues are depicted in blue; conserved phenylalanine and tyrosine residues are indicated in red. The plot was produced using WebLogo v3.7.4.[3]

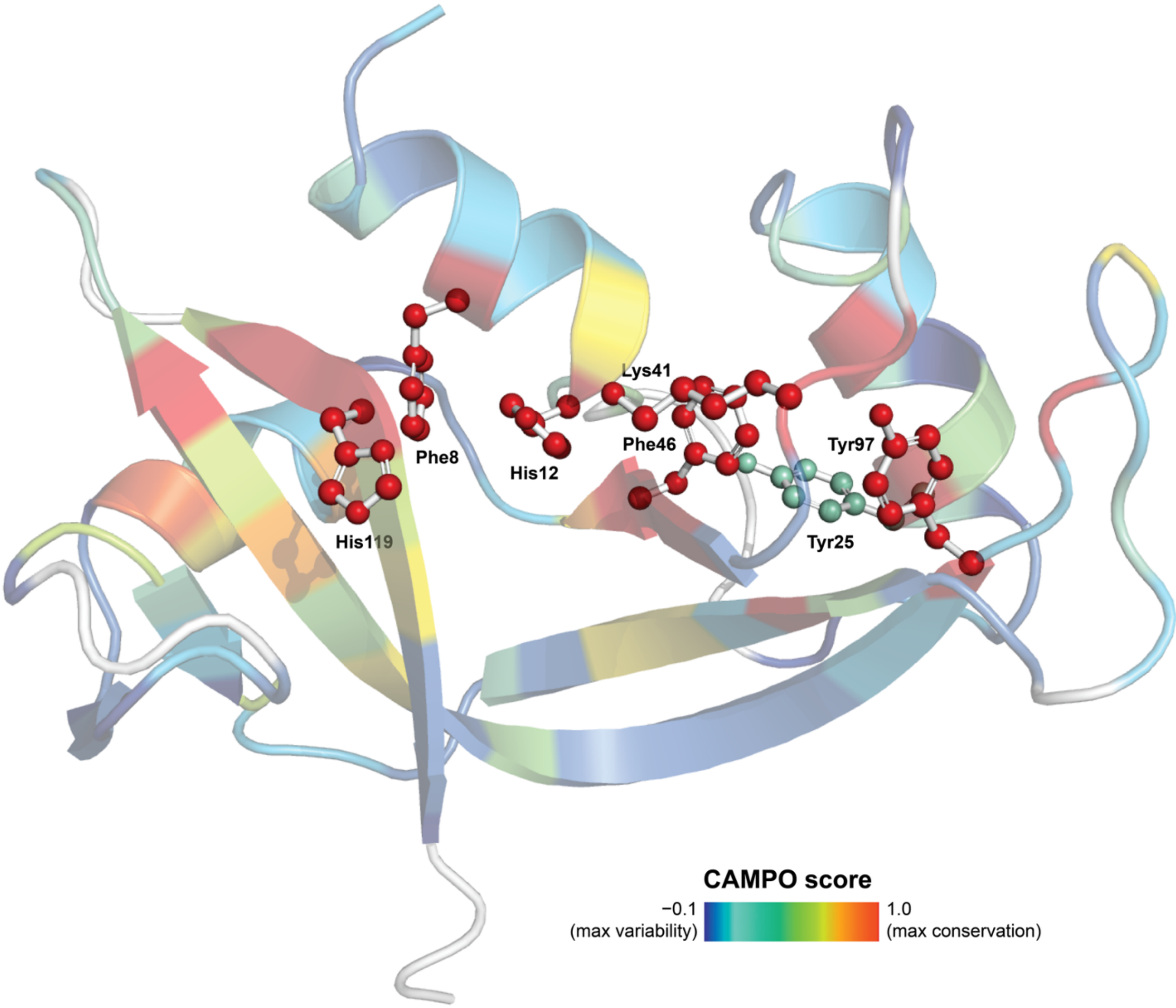


**Figure S5.** Structure of human RNase 1 (PDB entry 1Z7X[5]) depicting the conservation of residues in 18 homologs.[12] The side chains of aromatic residues within 7 Å of a methionyl sulfur atom and active-site residues His12, Lys41, and His119 are shown in ball-and-stick format.

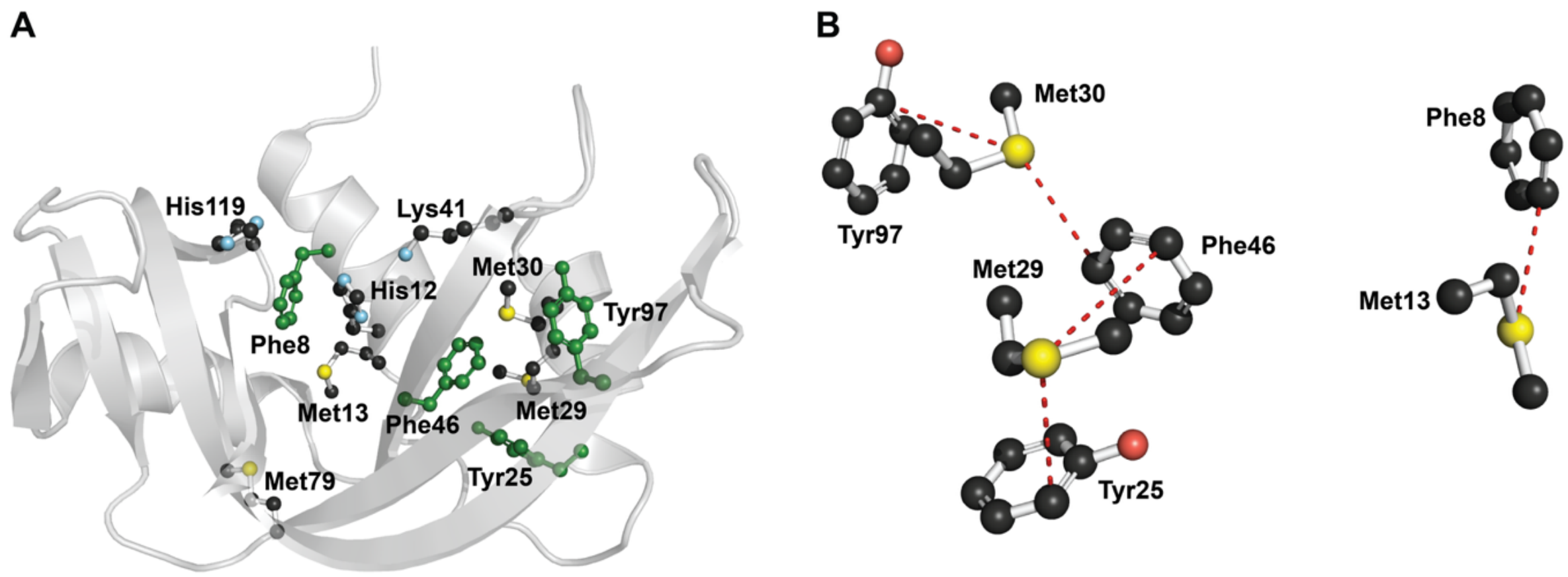


| Interaction | *d* (Å) | *θ* (°) | Sulfur orientation |
|---|---|---|---|
| Met13–Phe8 | 4.2 | 28.2 | Toward |
| Met29–Tyr25 | 4.4 | 70.4 | Away |
| Met29–Phe46 | 5.4 | 83.5 | Away |
| Met30–Phe46 | 4.0 | 27.9 | Toward |
| Met30–Tyr97 | 4.7 | 82.0 | Away |

**Figure S6.** Bovine RNase A *S*–aryl motifs. (A) Structure of RNase A (PDB entry 7RSA[18]) with active site, methionine, and aromatic residues engaged in *S*–aryl motifs represented in ball-and-stick format (active-site and methionine residues in CPK color scheme; aromatic residues in green). (B) Detailed representation of RNase A *S*–aryl motifs, indicated by red dashed lines (methionyl sulfur to the closest aromatic residue side-chain carbon). Only methionine side-chain and aromatic ring atoms are shown. The distance (*d*), approach angle (*θ*), and sulfur orientation for each *S*–aryl motif are listed.

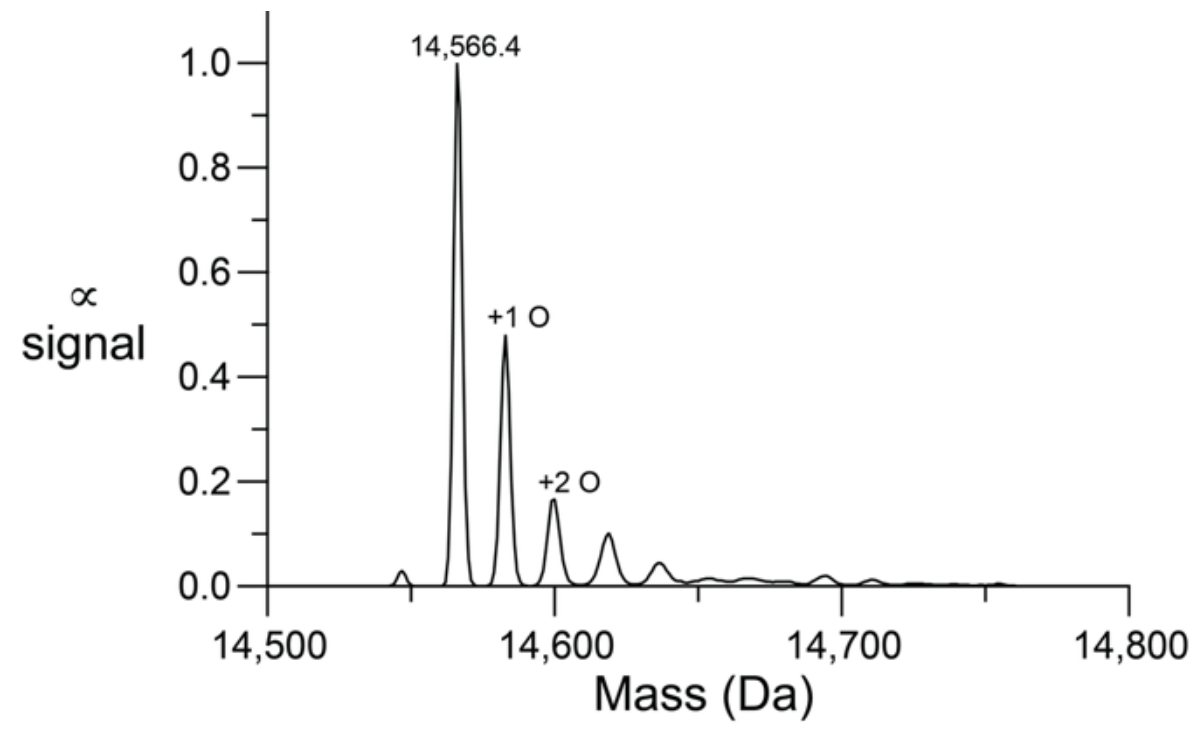


**Figure S7.** Deconvoluted Q-TOF mass spectrum of wild-type human RNase 1, purified by FPLC after the cleavage of recombinant M–DDDDK–RNase 1 by enterokinase to remove the N-terminal translation initiator methionine. Expected MW: 14,566.2 Da (UniProt ID P07998). Each oxidation event corresponds to $\Delta m$: +16.0 Da.

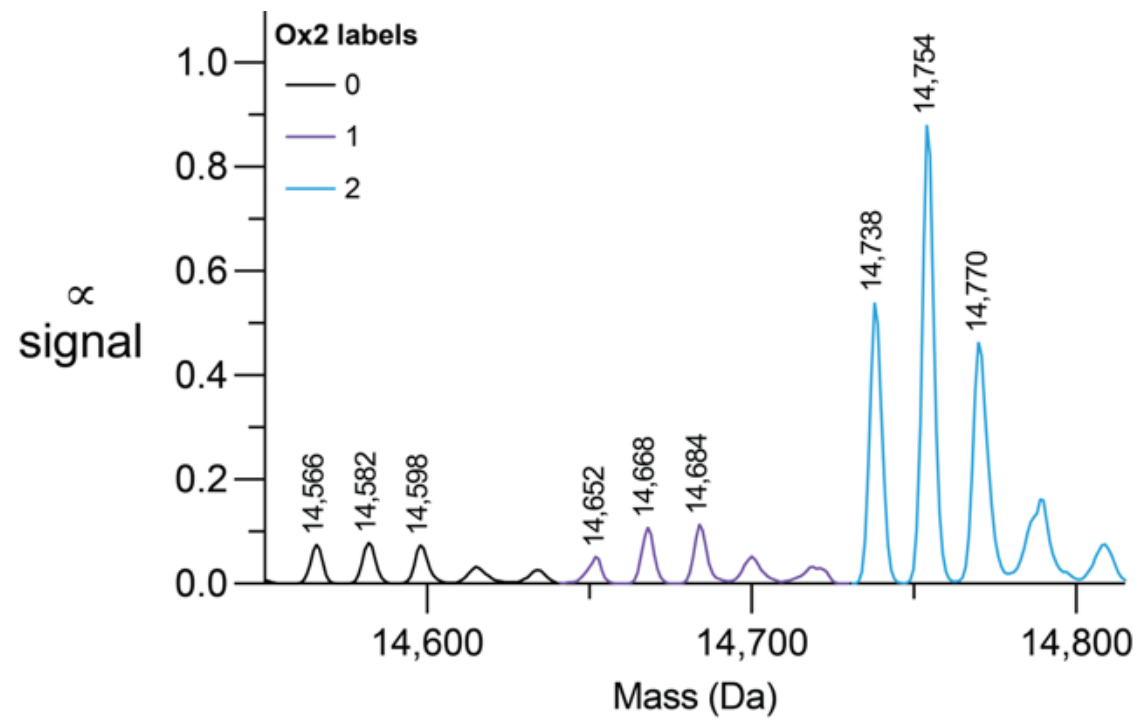


**Figure S8.** Inset of Figure 3A showing deconvoluted Q-TOF mass spectrum of Ox2-labeled RNase 1. Each oxidation event corresponds to $\Delta m$: +16.0 Da; each Ox2-labeling corresponds to $\Delta m$: +86.1 Da.

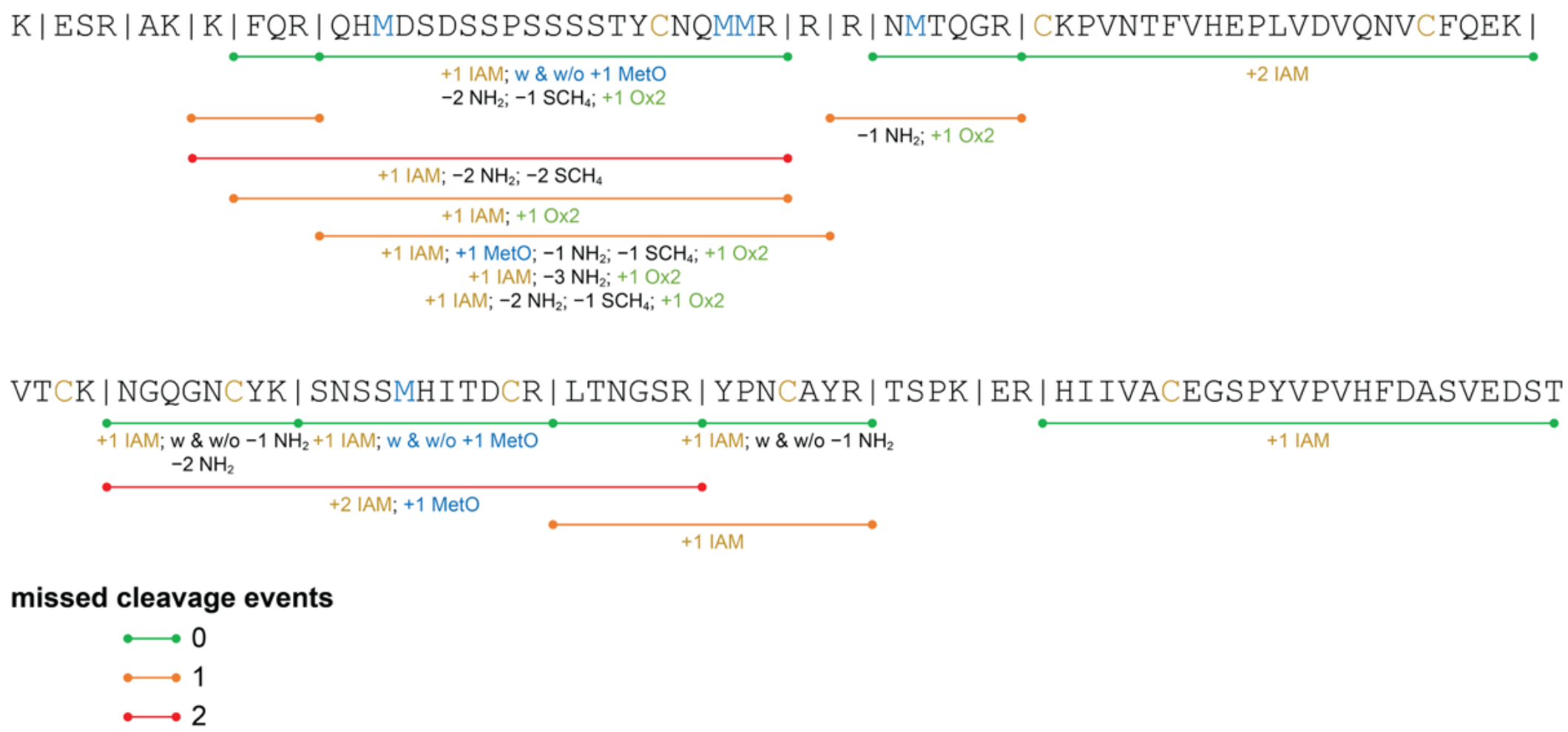


**Figure S9.** Tryptic peptide mapping for Ox2-labeled RNase 1, analyzed by Q-TOF mass spectrometry. Colored lines beneath the RNase 1 sequence indicate detected peptides (green: no missed tryptic cleavage; orange: one missed cleavage; red: two missed cleavages), with each line of text indicating a unique peptide and its predicted modifications. +IAM, cysteine alkylation with iodoacetamide; +MetO, methionine oxidation; +Ox2, methionine labeling with Ox2; $-NH_2$, asparagine/glutamine deamidation; $-SCH_4$, methionine dethiomethylation.[16] Peptides were detected at a $\Delta$10 ppm cut-off. Sequence coverage: 90.6%.

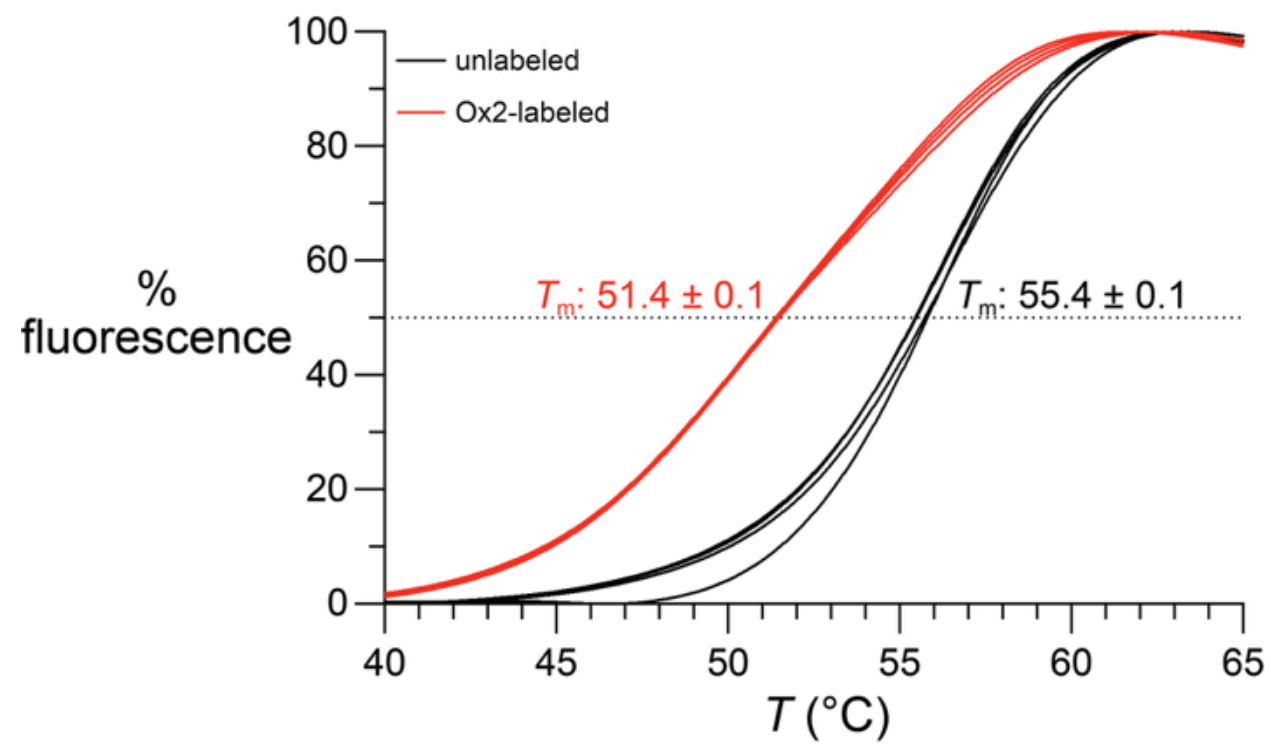


**Figure S10.** Thermal denaturation curves of unlabeled and Ox2-labeled RNase 1 in DPBS. $\Delta T_m = -4.0 \pm 0.1$ °C (mean ± SEM, $n = 4$).

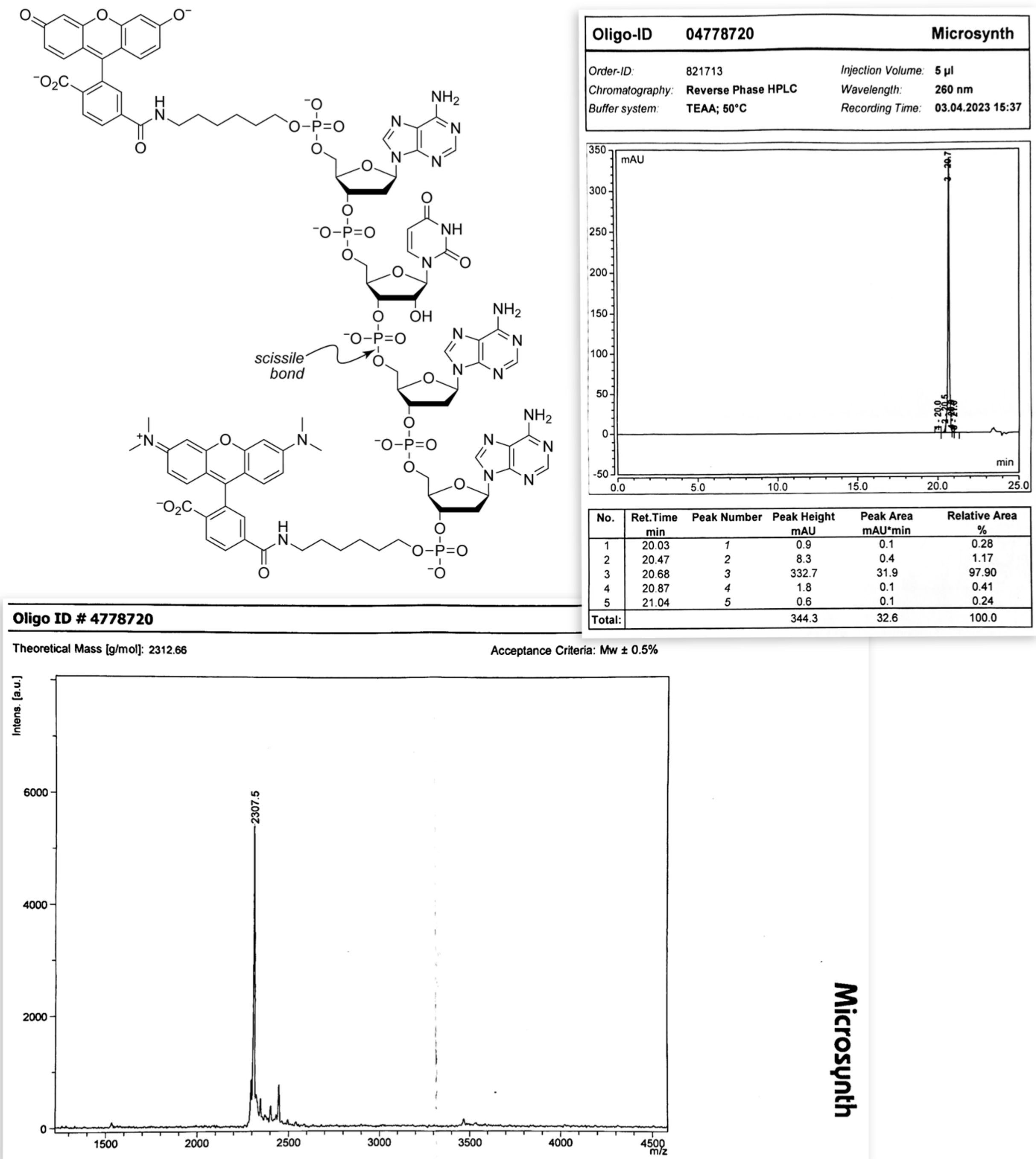


| No. | Ret.Time min | Peak Number | Peak Height mAU | Peak Area mAU*min | Relative Area % |
|---|---|---|---|---|---|
| 1 | 20.03 | 1 | 0.9 | 0.1 | 0.28 |
| 2 | 20.47 | 2 | 8.3 | 0.4 | 1.17 |
| 3 | 20.68 | 3 | 332.7 | 31.9 | 97.90 |
| 4 | 20.87 | 4 | 1.8 | 0.1 | 0.41 |
| 5 | 21.04 | 5 | 0.6 | 0.1 | 0.24 |
| Total: | | | 344.3 | 32.6 | 100.0 |

**Figure S11.** Structure of 6-FAM–dArU(dA)$_2$–6-TAMRA, which is the fluorogenic substrate for assays of ribonucleolytic activity and has only one scissile phosphodiester bond. The cleavage of that bond increases the fluorescence of the FAM moiety with $\lambda_{ex}$ = 493 ± 5 nm and $\lambda_{em}$ = 515 ± 5 nm. Insets: reversed-phase HPLC chromatogram (top) and MALDI-TOF mass spectrum (bottom) of synthetic 6-FAM–dArU(dA)$_2$–6-TAMRA, which was obtained from Microsynth (Balgach, Switzerland).

## S13. Supplementary Tables S1 and S2

**Table S1. Methionine conservation parameters in RNase 1 and 18 homologs**

| Residue | Conservation | Distance to… (Å)[a] | | | Frequency Exposed[b] |
|---|---|---|---|---|---|
| | | His12 | Lys41 | His119 | |
| Met13 | 6/19 | 10 ± 1 | 14 ± 1 | 15 ± 2 | 5/6 |
| Met29 | 11/19 | 13 ± 1 | 14 ± 1 | 20 ± 1 | 10/11 |
| Met30 | 19/19 | 9 ± 2 | 7 ± 1 | 16 ± 2 | 3/19 |
| Met35 | 7/19 | 6 ± 1 | 4 ± 1 | 12 ± 1 | 7/7 |
| Met79 | 7/19 | 15 ± 5 | 19 ± 4 | 15 ± 1 | 6/7 |

[a]Distances are from either Met $S^{\delta}$ to His $C^{\varepsilon 1}$ or Met $S^{\delta}$ to Lys $C^{\varepsilon}$, and are the mean ± SD ($n$ = number of conserved methionine residues). [b]Exposure is determined by solvent accessibility based on consensus criteria.[10,11]

**Table S2. Yield of [Nle]RNase 1 during folding and purification.**

| Step | Resultant Mass (μg) | % of Starting Mass | Protein State |
|---|---|---|---|
| Dissolution | 3100 | 100.0 | Dissolved; fully reduced polypeptide |
| Folding | 2620 | 84.5 | Oxidatively folded protein; mixture of native and nonnative forms |
| HPLC | 1001 | 32.3 | Purified; native protein in mixed solvent |
| Buffer exchange | 754 | 24.3 | Purified; native protein in aqueous buffer |

# S14. $^{1}H$ NMR Spectrum of Ox2

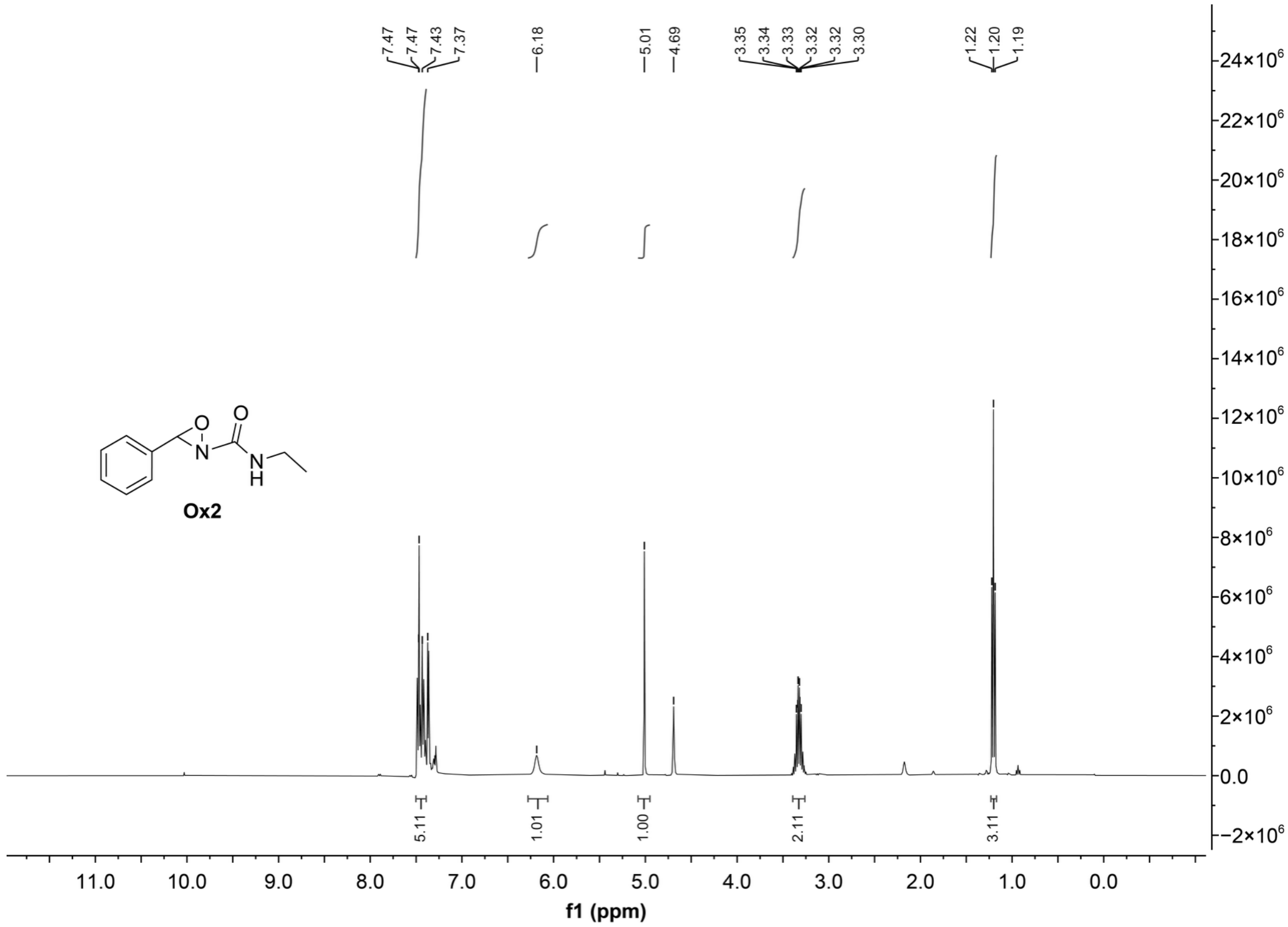

## S15. References


[1] N. Hartrampf, A. Saebi, M. Poskus, et al., "Synthesis of Proteins by Automated Flow Chemistry," *Science* 368 (2020): 980–987.

[2] A. Dereeper, V. Guignon, G. Blanc, et al., "Phylogeny.fr: Robust Phylogenetic Analysis for the Non-Specialist," *Nucleic Acids Research* 36 (2008): W465–W469.

[3] G. E. Crooks, G. Hon, J.-M. Chandonia, and S. E. Brenner, "WebLogo: A Sequence Logo Generator," *Genome Research* 14 (2004): 1188–1190.

[4] L. Willard, A. Ranjan, H. Zhang, et al., "WebLogo: A Sequence Logo Generator," *Nucleic Acids Research* 31 (2003): 3316–3319.

[5] R. J. Johnson, J. G. McCoy, C. A. Bingman, G. N. Phillips Jr., and R. T. Raines, "Inhibition of Human Pancreatic Ribonuclease by the Human Ribonuclease Inhibitor Protein," *Journal of Molecular Biology* 368 (2007): 434–449.

[6] L. Holm, A. Laiho, P. Törönen, and M. Salgado, "DALI Shines a Light on Remote Homologs: One Hundred Discoveries," *Protein Science* 32 (2023): e4519.

[7] G. Janson and A. Paiardini, "PyMod 3: A Complete Suite for Structural Bioinformatics in PyMOL," *Bioinformatics* 37 (2020): 1471–1472.

[8] R. C. Edgar, "Muscle5: High-Accuracy Alignment Ensembles Enable Unbiased Assessments of Sequence Homology and Phylogeny," *Nature Communications* 13 (2022): 6968.

[9] K. Uchida, "Histidine and Lysine as Targets of Oxidative Modification," *Amino Acids* 25 (2003): 249–257.

[10] S. M. Marino and V. N. Gladyshev, "Cysteine Function Governs Its Conservation and Degeneration and Restricts Its Utilization on Protein Surfaces," *Journal of Molecular Biology* 404 (2010): 902–916.

[11] J. C. Aledo, F. R. Cantón, and F. J. Veredas, "Sulphur Atoms from Methionines Interacting with Aromatic Residues are Less Prone to Oxidation," *Scientific Reports* 5 (2015): 16955.

[12] A. Paiardini, F. Bossa, and S. Pascarella, "CAMPO, SCR_FIND and CHC_FIND: A Suite of Web Tools for Computational Structural Biology," *Nucleic Acids Research* 33 (2005): W50–W55.

[13] T. J. Rutkoski, E. L. Kurten, J. C. Mitchell, and R. T. Raines, "Disruption of Shape-Complementarity Markers to Create Cytotoxic Variants of Ribonuclease A," *Journal of Molecular Biology* 354 (2005): 41–54.

[14] S. Lin, X. Yang, S. Jia, et al., "Redox-Based Reagents for Chemoselective Methionine Bioconjugation," *Science* 355 (2017): 597–602.

[15] K. P. Nandhini, M. Alhassan, C. G. L. Veale, F. Albericio, and B. G. De La Torre, "Methionine-Containing Peptides: Avoiding Secondary Reactions in the Final Global Deprotection," *ACS Omega* 8 (2023): 15631–15637.

[16] F. M. Lagerwerf, M. van de Weert, W. Heerma, and J. Haverkamp, "Identification of Oxidized Methionine in Peptides," *Rapid Communications in Mass Spectrometry* 10 (1996): 1905–1910.

[17] B. R. Kelemen, T. A. Klink, M. A. Behlke, S. R. Eubanks, P. A. Leland, and R. T. Raines, "Hypersensitive Substrate for Ribonucleases," *Nucleic Acids Research* 27 (1999): 3696–3701.

[18] A. Wlodawer, L. A. Svensson, L. Sjoelin, and G. L. Gilliland, "Structure of Phosphate-Free Ribonuclease A Refined at 1.26 Å," *Biochemistry* 27 (1988): 2705–2717.
